# Analysis of Bacterial Communities on North Sea Macroalgae and Characterization of the Isolated Planctomycetes *Adhaeretor mobilis* gen. nov., sp. nov., *Roseimaritima multifibrata* sp. nov., *Rosistilla ulvae* sp. nov. and *Rubripirellula lacrimiformis* sp. nov.

**DOI:** 10.3390/microorganisms9071494

**Published:** 2021-07-13

**Authors:** Sandra Wiegand, Patrick Rast, Nicolai Kallscheuer, Mareike Jogler, Anja Heuer, Christian Boedeker, Olga Jeske, Timo Kohn, John Vollmers, Anne-Kristin Kaster, Christian Quast, Frank Oliver Glöckner, Manfred Rohde, Christian Jogler

**Affiliations:** 1 Department of Microbiology, Radboud University, 6525 AJ Nijmegen, The Netherlands; sandra.wiegand@kit.edu (S.W.); n.kallscheuer@science.ru.nl (N.K.); ti.kohn@t-online.de (T.K.); 2 Institute for Biological Interfaces 5 (IBG-5), Karlsruhe Institute of Technology, 76131 Karlsruhe, Germany; john.vollmers@kit.edu (J.V.); anne-kristin.kaster@kit.edu (A.-K.K.); 3 Leibniz Institute DSMZ, 38124 Braunschweig, Germany; raspat88@gmail.com (P.R.); ast11@dsmz.de (A.H.); c.boedeker.dsmz@gmail.com (C.B.); olga.jeske@web.de (O.J.); 4 Institute of Bio- and Geosciences, Biotechnology (IBG-1), Forschungszentrum Jülich GmbH, 52428 Jülich, Germany; 5 Department of Microbial Interactions, Institute of Microbiology, Friedrich-Schiller University, 07743 Jena, Germany; mareike@jogler.de; 6 Max Planck Institute for Marine Microbiology, 28359 Bremen, Germany; c.quast@jacobs-university.de; 7 Alfred Wegener Institute Bremerhaven, MARUM, University of Bremen, 28359 Bremen, Germany; frank.oliver.gloeckner@awi.de; 8 Central Facility for Microscopy, Helmholtz Centre for Infection Research, 38124 Braunschweig, Germany; Manfred.Rohde@helmholtz-hzi.de

**Keywords:** budding bacteria, *Pirellulaceae*, *Lacipirellulaceae*, marine biofilms, epiphytic community, macroalgae, *Fucus* sp., *Ulva* sp., *Laminaria* sp.

## Abstract

Planctomycetes are bacteria that were long thought to be unculturable, of low abundance, and therefore neglectable in the environment. This view changed in recent years, after it was shown that members of the phylum *Planctomycetes* can be abundant in many aquatic environments, e.g., in the epiphytic communities on macroalgae surfaces. Here, we analyzed three different macroalgae from the North Sea and show that *Planctomycetes* is the most abundant bacterial phylum on the alga *Fucus* sp., while it represents a minor fraction of the surface-associated bacterial community of *Ulva* sp. and *Laminaria* sp. Especially dominant within the phylum *Planctomycetes* were *Blastopirellula* sp., followed by *Rhodopirellula* sp., *Rubripirellula* sp., as well as other *Pirellulaceae* and *Lacipirellulaceae*, but also members of the OM190 lineage. Motivated by the observed abundance, we isolated four novel planctomycetal strains to expand the collection of species available as axenic cultures since access to different strains is a prerequisite to investigate the success of planctomycetes in marine environments. The isolated strains constitute four novel species belonging to one novel and three previously described genera in the order *Pirellulales*, class *Planctomycetia*, phylum *Planctomycetes*.

## 1. Introduction

In aquatic environments, microorganisms either follow a planktonic lifestyle, floating or swimming in the water column, or switch to an attached state by forming biofilms on all kinds of natural or artificially introduced surfaces, e.g., steel and microplastics [1,2,3]. While biotic surfaces as such provide a source of organic matter, the same is also true for abiotic surfaces as soon as polar and hydrophobic materials start to accumulate on these submerged surfaces [3]. In general, marine biofilms are most often colonized by members of the marine *Roseobacter* clade (*Alphaproteobacteria*) as well as *Alteromonadaceae*, *Vibrionaceae* (both *Gammaproteobacteria*), and *Bacteroidetes* [3,4]. It is known that (seasonal) environmental dynamics, such as changes of temperature or salinity, but also stress factors, such as the presence of oxidants or metal ions, strongly influence epiphytic communities [5,6,7,8]. It was also shown that these communities can be very specific to their hosts [9,10].

A large fraction of marine biotic surfaces is formed by macroalgae, a generic term for sessile *Rhodophyta*, *Phaeophyta*, and *Chlorophyta* (red, brown, and green algae, respectively). Bacterial biofilms and marine macroalgae have been described to have a mutualistic as well as an opportunistic relation, with microorganisms protecting the alga from fouling-inducing organisms, enabling substrate exchange of, e.g., vitamin B_12_, fixing nitrogen, playing an important part in the algal morphology development, and influencing the release and settlement of algal spores, but also exploiting the rich repertoire of substrates on the algal surface [11,12,13]. Several studies on the composition of the various macroalgal epiphytic communities found *Alphaproteobacteria*, *Gammaproteobacteria*, and *Bacteroidetes* to often be the most dominant taxa within these communities, followed by—depending on the analyzed algae—*Actinobacteria*, *Firmicutes*, *Verrucomicrobia*, *Deltaproteobacteria*, and *Planctomycetes* [13,14,15,16,17,18]. Interestingly, *Aquificae*, *Chlorobi*, *Dyctioglomi*, *Lentisphaerae*, *Tenericutes*, and *Gemmatimonadetes* were only found on certain macroalgal groups [12].

Marine biofilms provide a rich source for hidden microbial diversity and functionalities, e.g., the synthesis of secondary metabolites [18]. The presence of members of the phylum *Planctomycetes* has been shown especially for *Ulva* sp., *Chondrus* sp., *Fucus* sp., and *Laminaria* sp. [19,20,21,22,23]. Usually, members of the class *Planctomycetia* were found to constitute the majority of the identified planctomycetes, followed by members of the OM190 lineage [19,21,22]. Nevertheless, the first two described planctomycetal isolates from macroalgae belong to the class *Phycisphaerae* [24,25,26]. In recent years, single-cell genomes [27] and axenic cultures from macroalgae were also obtained for the families *Pirellulaceae* [28,29,30,31], *Lacipirellulaceae* [32], *Planctomycetaceae* [33,34,35], and *Isosphaeraceae* [36].

Planctomycetes possess several traits that make them well-suited for the life in marine biofilms, for example the presence of a holdfast structure that facilitates surface colonization [3,25]. Species of the class *Planctomycetia* divide by budding, a feature they share with other frequent biofilm dwellers of the families *Hyphomonadaceae* and *Rhodobacteraceae* [25]. Despite the capability to quickly adapt to environmental changes [37], planctomycetes are described to be slow-growing organisms with generation times of several hours up to days, which makes them susceptible to being outgrown by other bacteria [25]. Thus, planctomycetes must apply other strategies to succeed, such as their big genomes that encode, e.g., a plethora of sulfatases enabling the degradation of complex sulfated polysaccharides found in the cell walls of various algae [38]. In addition, they possess many biosynthetic gene clusters that are potentially involved in the production of secondary metabolites [39], e.g., a novel group of small molecules that is responsible for altering the species composition of biofilms [40]. An additional advantage over the use of exoenzymes for degradation might also be their capability to accumulate complex carbohydrates such as dextran in their enlarged and invaginated periplasmic space [41]. Taken together, the recent findings point towards complex allelopathic interactions of planctomycetes with marine macroscopic phototrophs.

Here, we report the results of a 16S rRNA gene amplicon-based cultivation-independent analysis of bacterial communities of algal biofilms and the surrounding waters, elucidating the diversity and abundance of members of the phylum *Planctomycetes* in biofilms of the three North Sea macroalgal specimens (*Fucus* sp., *Ulva* sp., and *Laminaria* sp.). Moreover, we describe the physiology, chemotaxonomy, morphology, cell biology, genomic features, and phylogeny of four novel planctomycetal isolates, representing one novel genus and three novel species within this phylum.

## 2. Materials and Methods

### 2.1. Sampling and Sample Preparation

Algal material of *Laminaria* sp., *Fucus* sp., and *Ulva* sp. and samples from water surrounding each alga were collected in sterile 1 L bottles during low tide on the north shore of Helgoland Island, Germany on 5 June 2013 (exact sampling location 54.188° N, 7.875° E, water temperature 13 °C). The samples were immediately transferred to the Biologische Anstalt Helgoland. The algae were stored in separated natural seawater tanks at 13 °C and water samples were stored at 4 °C. All samples were transferred to the DSMZ, Braunschweig, Germany within 24 h and processed within 4 h after arrival. In the laboratory, 300 mL of the water samples were homogenized by gentle stirring and subjected to a two-step filtration process to separate aggregated/particle-attached and planktonic bacterial cells [42]. In the first step, the water was filtered through a borosilicate glass microfiber filter (Whatman GF/D, GE Healthcare, Dassel, Germany) with a pore size of 2.7 µm to retain aggregated cells and marine particles. The same 300 mL of water was then filtered through a polycarbonate membrane filter (Isopore/Merck, Darmstadt, Germany) with a pore size of 0.22 µm to obtain the fraction of planktonic microorganisms. Filters were stored at −20 °C. Material of *Laminaria* sp., *Fucus* sp., and *Ulva* sp. was gently rinsed two times with filter-sterilized (Corning bottle top filters, Sigma-Aldrich, Schnelldorf, Germany) natural seawater to remove unattached bacteria. Biofilm suspensions were prepared by carefully scraping off algal biofilms into sterile natural seawater with single-use scalpels. Biofilm suspensions of all three algae were homogenized by vortexing, split into two equal volumes and stored at −20 °C until DNA extraction or at 4 °C until use in cultivation experiments (on the same day). Biofilms used for cultivation were supplemented with 20 mg/mL cycloheximide to inhibit the growth of fungi. Algal material was rinsed two times with sterile natural seawater and treated with 20 mg/mL cycloheximide dissolved in sterile natural seawater. Ten pieces of the collected algae with a diameter of 3 cm were sampled with a sterilized metal puncher and stored in sterile natural seawater until cultivation later that day. For each piece, five smaller pieces (diameter 0.5 cm) directly adjacent to the larger pieces were gathered and directly fixed in 1.5% (*v/v*) formaldehyde solution for subsequent field emission scanning electron microscopy.

### 2.2. Isolation and Cultivation of Novel Strains

For bacterial cultivation, different media were prepared (Supplementary Table S1). All media contained 2.38 g/L HEPES (Serva, Heidelberg, Germany) as buffering agent, 20 mL/L mineral salt solution, 250 mL/L concentrated artificial seawater (ASW) and were adjusted to pH 8.0 with 5 M KOH. After sterilization by autoclaving, media were supplemented with 5 mL/L double concentrated vitamin solution and 1 mL/L trace element solution (for exact recipes of mineral salt, vitamin and trace element solution as well as ASW, see [39]. For the preparation of solid media, either 12 g/L agar (BD Difco, Franklin Lakes, NJ, USA), washed three times with deionized water, or 8 g/L gellan gum (Gelrite, Serva, Heidelberg, Germany) were autoclaved separately and added to the medium prior to pouring plates. For the initial isolation, 0, 20, or 100 mg/L cycloheximide were added to each medium to test the necessity for anti-fungal agents. In addition, media were also supplemented with 2000 mg/L carbenicillin or a mixture of 200 mg/L ampicillin and 1000 mg/L streptomycin to test the effectiveness of different antibiotic agents to selectively enrich planctomycetes (which show natural resistance to the used antibiotics). Initial isolation media also contained either 0.25 g/L peptone (BD Difco), 0.25 g/L yeast extract (BD Difco) and 10 mL/L of a 2.5% (*w*/*v*) glucose solution (M1H ASW medium) or 20 mL/L of a 5% (*w*/*v*) solution of *N*-acetyl-d-glucosamine (NAG ASW medium) as carbon and nitrogen sources. For the subsequent cultivation of novel strains, all media contained peptone, yeast extract and NAG as additional components (M1H NAG ASW) (Supplementary Table S1).

Algal attachment experiments were performed with media solely containing either 2 g/L *Ascophyllum nodosum* (BioOrigins, Sandleheath, United Kingdom) or *Fucus serratus* (Mountain Fresh Health Foods, Twin Falls, ID, USA) powder. Biofilm suspensions and algal portions of *Laminaria* sp., *Fucus* sp., and *Ulva* sp. were prepared as described above and used to inoculate liquid and solid M1H ASW and NAG ASW medium (Supplementary Table S1). Solid media were inoculated either with 15 µL biofilm suspension (spread out with glass beads) or one algal piece, which was swabbed over the plate and then placed in the middle. In addition, 100 mL liquid medium (M1H ASW medium and NAG ASW medium) were inoculated with either 100 µL biofilm suspension or with one round algal piece. Addition of antibiotics served as selection pressure to enrich planctomycetes, which often turn out to be resistant to several antimicrobial agents, e.g., β-lactam antibiotics [43,44]. A floating filter cultivation technique was adapted from a previously published protocol [45]: 6-well inoculation plates (Corning Costar cell culture plates, Sigma-Aldrich) were filled with 5 mL of either M1H ASW or NAG ASW medium, supplemented with 100 mg/L cycloheximide and either 2000 mg/L carbenicillin or 1000 mg/L streptomycin. For inoculation, biofilm suspensions obtained from the three different algae were diluted 1:5, 1:10, and 1:50 in 5 mL sterile seawater and filtered through black polycarbonate filters (0.1 µm retention size, diameter 25 mm, GE Osmonics, Minnetonka, MN, USA). The filters were then placed to float on the medium. All cultures were incubated in the dark at 20 °C until colony growth or change in cell density (enrichment cultures) was observed. Cell densities were measured as optical density at 600 nm (OD_600_) using an Ultrospec II spectrophotometer (LKB Biochrom, Cambridge, United Kingdom). Colonies with certain phenotypes, e.g., a pink- or cream-colored pigmentation, smooth colony appearance and slow growth were replated. Enrichment cultures were checked for the enrichment of bacteria with characteristic traits of planctomycetes, such as polar budding or pear- to spherical cell shape [25], by wide-field microscopy and were subsequently plated on solid medium. Strains were identified by 16S rRNA gene sequencing as previously described [46] and replated three times for purification after having confirmed that the strains are indeed members of the phylum *Planctomycetes*. Isolates with 16S rRNA gene sequence identity values below 97% to any then-described isolate were considered as novel. Out of all strains isolated by the used methods, four strains (K22.7^T^, FF011L^T^, HG15A2^T^ and EC9^T^) were subjected to a more detailed characterization.

### 2.3. Determination of Temperature and pH Optima for Growth

Strains K22.7^T^, FF011L^T^, HG15A2^T^, and EC9^T^ were grown in M1H NAG ASW medium to the early stationary phase. This pre-culture was then used to inoculate medium (1:10 dilution) for determination of the temperature and pH optima for growth. For the determination of the optimal temperature for growth, temperatures of 10, 12, 14, 16, 18, 20, 22, 24, 26, 28, 32, 34, 36, and 40 °C were tested in duplicates. For the determination of the optimal pH, M1H NAG ASW medium buffered with MES, HEPES, HEPPS or CHES (10 mM final concentration) was used over a pH range from 5–10 in 0.5 steps. Cell densities were measured as optical density at 600 nm (OD_600_). Growth rates were obtained from the plot of ln(OD_600_) against the cultivation time.

### 2.4. Catalase and Cytochrome Oxidase Activity

Catalase activity was determined by reaction of cell material obtained from an exponentially growing culture with 3% (*v*/*v*) H_2_O_2_ solution, resulting in the release of oxygen (catalase-positive). Cytochrome oxidase activity was determined using Bactident Oxidase test stripes (Merck Millipore, Darmstadt, Germany) following the manufacturer’s instructions.

### 2.5. Substrate Utilization

Substrate utilization of strains K22.7^T^, FF011L^T^, HG15A2^T^, and EC9^T^ was determined using the Biolog GN2 MicroLog test panel for Gram-negative bacteria in duplicates. Sterile glass tubes were prepared in duplicates with a basic sterile medium mixture containing 14.2 mL IF-0a inoculation fluid (Biolog, Hayward, CA, USA), 1.6 mL of a 10× salt solution (containing per liter: 200 g NaCl, 40 g Na_2_SO_4_, 30 g MgCl_2_·6H_2_O, 5 g KCl, 2.5 g NH_4_Cl, 2 g KH_2_PO_4_, 1.5 g CaCl_2_·2H_2_O), 160 µL 1 M HEPES buffer (pH 8.0), 80 µL double concentrated vitamin solution, and 16 µL trace element solution (for recipes, see [47]). Tubes were inoculated with bacterial colony material from exponentially growing cultures to a turbidity of 50–60% transmittance using a turbidimeter (AES Chemunex BLG 3531). To enable the comparison of utilization values, the data of each single experiment were normalized to 100. Only results >0 were taken into consideration. A heatmap of resulting values was computed using the R environment [48] with the heatmap.2() function of the gplots package, v. 3.1.0.

### 2.6. Cellular Fatty Acid Analysis

Biomass of the isolated strains was obtained from liquid cultures grown in M1H NAG ASW medium (Supplementary Table S1) at the respective optimal growth temperature until the stationary phase was reached. Cells of 50 mL culture were harvested by centrifugation at 10,000× *g* for 20 min (Avanti Centrifuge I-26 XPI, Beckman Coulter, Krefeld, Germany) and the supernatant was discarded. Then, 30 mg of lyophilized biomass were analyzed by the Identification Service of the German Collection of Microorganisms and Cell Cultures (DSMZ) according to the standard protocols of the facility based on previously published methods [49,50].

### 2.7. Algal Attachment Assay

To investigate the attachment and growth behavior of strain K22.7^T^, which was isolated from *Fucus* sp., an artificial marine snow (AMS) medium containing algal powder from the brown alga *Fucus serratus* as sole carbon and nitrogen source was prepared. Since the AMS medium was very dark in color and contained a lot of algal particles, OD_600_ determination for assessing the growth of the planctomycete was not possible. 

### 2.8. DNA Isolation and Amplification for Sequence-Based Community Analysis

DNA of algal biofilms and water filters was extracted using the PowerBiofilm DNA Isolation Kit (MoBio Laboratories, Carlsbad, CA, USA) following the manufacturer’s protocol with a few exceptions: incubation at 37 °C in buffer B1 was increased to an overnight step; incubation at 55 °C was increased to 60 min; incubation at 4 °C was increased to 20 min. Bead-beating was performed in a FastPrep-24 instrument (MP Biomedicals, Eschwege, Germany) at 5.5 m/s for 30 s. DNA was eluted in 100 µL BF7 buffer and stored at −20 °C until further processing. The DNA was quantified using the Qubit dsDNA HS or BR Assay Kits (Thermo Fisher Scientific, Dreieich, Germany). Genomic DNA was amplified by multiple displacement amplification (MDA) based on phage Φ29 (phi29) DNA polymerase [51]. For this purpose, the Illustra GenomiPhi V3 DNA Amplification Kit (GE Healthcare, Solingen, Germany) [52] was used following the general recommendations of the manufacturer. For one single amplification reaction (20 µL total volume), 1 ng of genomic DNA was used. To reduce remaining stochastic amplification bias, three independent reactions per sample were pooled. To reduce contamination with external DNA, preparation steps not involving a DNA template were performed in a PCR cabinet (AirCleanSystems, StarLab, Hamburg, Germany) previously decontaminated using DNA-away (Molecular BioProducts, Thermo Fisher Scientific, Dreieich, Germany) and UV light for 1 h in a no-template room. DNA was added in another low-template room in a second PCR cabinet and amplification reactions were performed in a thermal cycler (Veriti 96-Well, Applied Biosystems, Waltham, MA, USA). MDA-amplified DNA was stored at −20 °C until further processing.

Amplification of the variable region 3 (V3) of 16S rRNA genes was performed using two subsequent PCR amplifications. The first protocol was used to enrich the V3 region of MDA-obtained DNA. In this protocol, the universal forward primer 341f (5′-CCT ACG GGW GGC WGC AG-3′) and the reverse primer uni515r (5′-CCG CGG CTG CTG GCA C-3′) (modified from 518r) [53] were used. The second PCR protocol was then performed with extended V3 region primers V3F (5′-AAT GAT ACG GCG ACC ACC GAG ATC TAC ACT CTT TCC CTA CAC GCT CTT CCG ATC TCC TAC GGG WGG CWG CAG-3′) and indexed V3R primers (5′-CAA GCA GAA GAC GGC ATA CGA GAT XXX XXX GTG ACT GGA GTT CAG ACG TGT GCT CTT CCG ATC TCC GCG GCT GCT GGC AC-3′) (modified from [54]). Sample/index combinations are listed in Supplementary Table S2. PCR reactions for the first protocol of 50 µL contained 25–29 µL microbial DNA-free water (Qiagen, Hilden, Germany), 10 µL 5× Q5 Reaction Buffer (New England Biolabs, Frankfurt am Main, Germany), 10 µL 5× Q5 High GC Enhancer (New England Biolabs), 1 µL dNTP Mix (final concentration 200 µM, New England Biolabs), 0.5 µL of each primer (341f and uni515r, final concentration 0.1 µM), 0.5 µL Q5 High fidelity DNA Polymerase (final concentration 0.02 U/µL, New England Biolabs) and 1–5 µL MDA-amplified gDNA (~500 ng). The cycling program consisted of an initial denaturation step at 94 °C, 5 min, followed by 10 cycles of denaturation at 94 °C, 1 min, annealing at 63 °C, 1 min, elongation at 72 °C, 1 min, and a final elongation step at 72 °C, 10 min. Three independent pre-amplification reactions were pooled and stored at 4 °C until further processing. The next PCR amplification was performed to add sequence indices and adapter sequences for subsequent Illumina sequencing. PCR reactions of 50 µL contained 13.1 µL PCR-grade H_2_O (Qiagen, Hilden, Germany), 10 µL 5× Q5 Reaction Buffer (New England Biolabs), 10 µL 5× Q5 High GC Enhancer (New England Biolabs), 1 µL dNTP Mix (final concentration 200 µM, New England Biolabs), 0.2 µL of each primer (V3F and V3R, final concentration 0.2 µM), 0.5 µL Q5 High fidelity DNA Polymerase (final concentration 0.02 U/µL, New England Biolabs) and 10 µL PCR product of the first PCR as DNA template. Amplification was performed with a cycling program including an initial denaturation step at 98 °C, 5 min, followed by 10 cycles of denaturation at 98 °C, 1 min, annealing at 65 °C, 1 min, elongation at 72 °C, 1 min and a final elongation step at 72 °C, 5 min. To reduce stochastic amplification bias, three independent amplifications were performed. Amplicon PCR products were separated by agarose gel electrophoresis (2% (*w/v*) agarose, Serva) in 1× TRIS-acetate-EDTA buffer (Applichem, Darmstadt, Germany) at 130 V for 90 min. The gel was stained with SYBR gold nucleic acid gel stain (Thermo Fisher Scientific) for 60 min and DNA was visualized by UV light. Amplicon bands (fragment size of ~300 bp) were cut out and extracted using the NucleoSpin Gel and PCR Clean-up kit (Macherey-Nagel, Dueren, Germany). All three replicates of each sample were purified over the same column.

### 2.9. Amplicon Sequencing and Sequence Processing

Reads of V3 amplicons obtained from Illumina multiplex sequencing (MiSeq) were quality trimmed using the tool Trimmomatic v. 0.36 [55] with the following arguments: “LEADING:3 TRAILING:3 SLIDINGWINDOW:4:15 MINLEN:105”. The trimmed sequences were then filtered for those starting with the forward and ending with the reverse primer. The remaining sequences were subsequently checked for chimera using UCHIME [56]. Non-chimeric sequences were further processed by clipping the forward and the reverse primer sequences and applying a length filter for sequences between 120 and 167 bp. Sequences below or above this cut-off were found to be chimeric sequences that were not detected by the UCHIME algorithm. The processed sequences were submitted to SILVAngs for taxonomic classification [57]. Files were uploaded as suggested in the SILVAngs user-guide and data were processed by the SILVAngs software according to the protocol, including alignment with the SINA aligner [58]. During the process, a de-replication step, eliminating 100% identical reads by only processing the longest read, as well as operational taxonomic unit (OTU) definition and clustering [59] were performed. OTUs were classified by a local BLAST search using blastn with default parameters in accordance with the non-redundant version of the SILVA SSU Ref database [57].

### 2.10. Wide Field Microscopy

Cells of the four isolated strains were immobilized on a 1% (*w*/*v*) agarose-pad in MatTek 35 mm glass-bottom dishes and imaged under phase-contrast illumination using a Nikon Eclipse Ti inverse microscope at 100-fold magnification and employing a Nikon DS-Ri2 camera. To determine the cell size of the novel strains, 100 individual cells of each strain were measured using the NIS-Elements software V4.3 (Nikon Instruments, Amsterdam, The Netherlands). Z-stacks were imaged to visualize algal attachment. To increase overall sharpness of phase contrast images, z-stacks were processed with the software PICOLAY (www.picolay.de, accessed on 15 June 2015) using the focus stacking option. By using this option, the program takes the sharp areas of each stack, generating a single overall sharp picture.

### 2.11. Field Emission Scanning Electron Microscopy (SEM) of Bacteria, Biofilms on Algal Pieces and Algae Granules

After cultivation of the isolated strain K22.7^T^ with *Fucus serratus* powder (Mountain Fresh, Health Foods, Twin Falls, ID, USA), algal particles and attached bacteria were fixed in modified HEPES buffer (3 mM HEPES, 0.3 mM CaCl_2_, 0.3 mM MgCl_2_, 2.7 mM sucrose, pH 6.9) containing 1% (*v*/*v*) formaldehyde for 1 h on ice and were washed once with the same buffer. The same protocol was used for the fixation of axenic cultures of the isolated planctomycetal strains. Cover slips with a diameter of 12 mm were coated with 50 µL of a 0.1% (*w*/*v*) poly-l-lysine solution (Sigma-Aldrich) for 10 min, washed with distilled water and air-dried. Algal pieces or 50 µL of the fixed bacteria solution were placed on a cover slip and allowed to settle for 10 min. Cover slips were then fixed in 1% (*v*/*v*) glutaraldehyde in TE buffer (20 mM TRIS, 1 mM EDTA, pH 6.9) for 5 min at room temperature and subsequently washed twice with TE buffer before dehydrating in a graded series of acetone (1.5 mL each of 10%, 30%, 50%, 70%, 90%, 100% (*v*/*v*)) on ice for 10 min at each concentration. Samples from the 100% acetone step were brought to room temperature before placing them in 20 mL fresh 100% acetone. Samples were then subjected to critical point drying with liquid CO_2_ (CPD 300, Leica). Dried samples were covered with a gold/palladium (80/20) film by sputter coating (SCD 500, Bal-Tec, Leica Biosystems, Wetzlar, Germany) before examination in a field emission scanning electron microscope (Zeiss Merlin, Jena, Germany) using the Everhart Thornley HESE2-detector and the inlens SE-detector in a 25:75 ratio at an acceleration voltage of 5 kV.

### 2.12. Transmission Electron Microscopy (TEM)

Thin sections of strains K22.7^T^, FF011L^T^, HG15A2^T^, and EC9^T^ were prepared by high pressure freezing and freeze substitution as previously described [60]. Sections were subsequently analyzed employing a JEOL 1200EX 80 kV TEM microscope.

### 2.13. Genome Information of the Isolated Strains

The genomes of all strains were published previously [39] and are available from RefSeq under accession numbers CP036261 (EC9^T^), CP036262 (FF011L^T^), CP036263 (HG15A2^T^) and CP036525 (K22.7^T^). The GenBank accession numbers of the respective 16S rRNA genes are MK554523, MK559974, MK559975, and MK559976.

### 2.14. Construction of Phylogenetic Trees

Maximum likelihood 16S rRNA gene sequence-based phylogeny was computed for the novel strains, all described planctomycetal species of the order *Pirellulales* [28,29,30,31,40,42,61,62,63,64,65,66,67,68,69,70,71,72,73] and an outgroup of three strains from the order *Planctomycetales* (acc. no. MK554524, MK554557, and X62911). The 16S rRNA gene sequences were aligned with SINA [58]. The phylogenetic analysis was then done by employing a maximum likelihood approach with 1000 bootstraps, the nucleotide substitution model GTR, gamma distribution and estimation of proportion of invariable sites (GTRGAMMAI option) [74].

For the multilocus sequence analysis (MLSA), the unique single-copy core genome of all analyzed genomes was determined with proteinortho5 [75] with the ‘selfblast’ option enabled, a coverage of 50%, and an e-value of 1e-05. The protein sequences of the resulting orthologous groups were aligned using MUSCLE v.3.8.31 [76]. After clipping, partially aligned *C*- and *N*-terminal regions and poorly aligned internal regions were filtered using Gblocks [77]. The final alignment of 570 ubiquitous genes with a combined length of 292,572 conserved amino acid residues was concatenated and clustered using FastTree [78]. The outgroup consisted of three genomes from strains of the order *Planctomycetales* (acc. no. CP036342, CP036347, and CP001744). Visualizations were performed with iToL v.4 [79].

Members of the novel genus *Roseiconus* [73] clustered within the earlier described genus *Stieleria* [40,62]. The results for both genera are therefore summarized under the latter term for all analyses.

### 2.15. Analysis of Phylogenetic Markers

The *rpoB* nucleotide sequences encoding the RNA polymerase β-subunit were taken from the respective, previously published genomes and the sequence identities were determined as described before [80] using Clustal Omega [81]. Alignment and matrix calculation were performed only using those parts of the sequence that would have been sequenced with the described primer set. The average nucleotide identity (ANI) was calculated using OrthoANI [82]. The average amino acid identity (AAI) was obtained with the aai.rb script of the enveomics collection [83] and the percentage of conserved proteins (POCP) was calculated as described before [84].

### 2.16. Analysis of Genome-Encoded Features

A genome-based analysis of encoded enzymes participating in central carbon metabolism was conducted by examining locally computed InterProScan [85] results cross-referenced with information from the UniProt [86] database and BLASTp results of typical protein sequences. The prediction of biosynthetic gene clusters for the analysis of secondary metabolites and small molecules was performed with antiSMASH 5 locally (enabled parameters: cb-general, cb-knownclusters, cb-subclusters, asf, pfam2go, smcog-trees, genefinding-tool prodigal) [87]. The analysis of the pan and core genomes was undertaken with anvi’o [88], following the pangenomics workflow [89].

## 3. Results

To explore the epiphytic bacterial communities of macroalgae found at the shore of Helgoland Island, the only German high seas island, we sampled organisms of three different genera, namely the brown algae *Laminaria* sp., *Fucus* sp., as well as the green alga *Ulva* sp. All specimens were conserved appropriately and subjected to cultivation-independent analyses and used for the cultivation of novel bacterial taxa.

### 3.1. Bacterial Communities of North Sea Algae Surfaces

**Figure 1:**
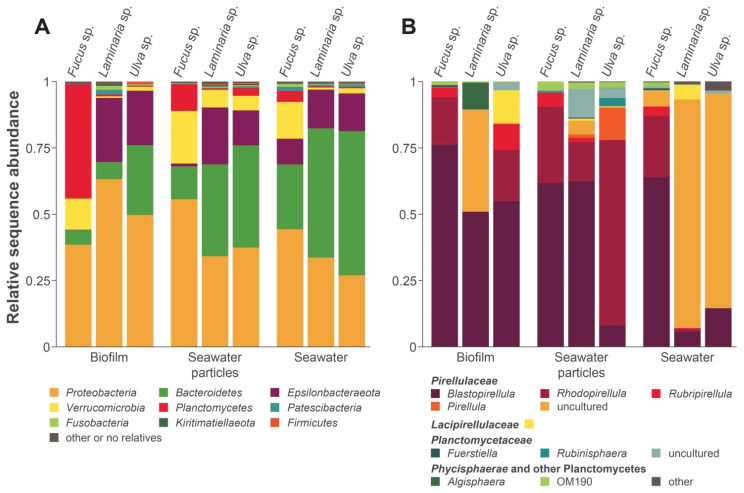
Bacterial epiphytic community on different macroalgae. The composition of the biofilms was analyzed based on 16S rRNA gene sequence-determined taxonomic classification on phylum level (**A**) and, for *Planctomycetes*, on genus level (**B**). The examined samples were taken from the algal biofilms and the surrounding waters which were subdivided in particles and aggregates (seawater particles) and planktonic bacteria (seawater). Taxa with an abundance <1% are summarized as “other (or no relative)”. The numbers of sequences, average length and numbers of operational taxonomic units are provided in Table S3.

In a first step, the bacterial communities associated with the macroalgae were investigated by amplification and sequencing of the 16S rRNA gene V3 region. Both, algal biofilms and surrounding waters were included in the analysis after the water samples were split into two fractions by filtration (2.7 µm filter retention size) to be able to target the planktonic bacteria and the surrounding aggregates and particles individually. For each of the nine samples (Figure 1), between 32,381 and 133,343 reads resulting in 546–1252 OTUs were obtained (Supplementary Table S3).

At the phylum level (Figure 1A), *Laminaria* sp. and *Ulva* sp. communities were rather similar despite the distant relation of the two host organisms. Both were dominated by *Proteobacteria* (63.2% and 49.7%, respectively). Two other major taxa were the *Bacteroidetes* (6.5% and 26.3%) and *Epsilonbacteraeota* (24.2% and 20.5%). Other lineages, including *Verrucomicrobia*, *Planctomycetes*, *Patescibacteria* (Candidate Phyla Radiation), *Fusobacteria*, *Kiritimatiellaeota*, and *Firmicutes*, were present, but each constituted <1.8% of the biofilms. In comparison with the *Laminaria* sp. and *Ulva* sp.-associated communities, the general composition of the surrounding particles and planktonic bacteria only changed slightly (Figure 1A): while the *Bacteroidetes* gained dominance over the *Proteobacteria*, *Verrucomicrobia* and *Planctomycetes* also became more prominent. In recent years, *Ulva* sp. biofilms have been analyzed in particular and were often shown to be primarily composed of *Alphaproteobacteria* and *Bacteroidetes*, often followed by *Gammaproteobacteria*, *Actinobacteria*, *Firmicutes*, *Cyanobacteria*, *Verrucomicrobia*, and *Planctomycetes* [8,10,12,14,15,16]. Our results are mostly consistent with these results, with exception to the high abundance of *Epsilonbacteraeota* and the absence of considerable amounts of *Actinobacteria* and *Cyanobacteria*. The identification of taxa such as *Patescibacteria* and *Kiritimatiellaeota* might be explained by the only recent work that was dedicated towards these clades and now enables their detection [90,91,92]. In contrast to other studies [10,16], the dominant *Proteobacteria* were not *Alphaproteobacteria*, but *Gammaproteobacteria*, which constituted between 78.7% and 90.3% of the total *Proteobacteria* and primarily included *Alteromonadales* (81.3% of *Gammaproteobacteria* on *Laminaria* sp.) or *Thiotrichales* (42.6% of *Gammaproteobacteria* on *Ulva* sp.), followed by *Oceanospirillales* and *Vibrionales*. Interestingly, *Gammaproteobacteria*, in particular taxa of the orders *Alteromonadales* and *Oceanospirillales*, were found to be highly abundant in young marine biofilms [93] and might be used as an indicator for the age of the examined biofilms.

The epiphytic bacterial community of *Fucus* sp. was significantly different to the other two macroalgae. The phylum *Planctomycetes* represents the dominant fraction of the bacterial community in the biofilm (43.1%), followed by *Proteobacteria* (38.5%), *Verrucomicrobia* (11.7%), and *Bacteroidetes* (5.6%). In the samples of the surrounding waters, *Planctomycetes* lost their high prevalence, while *Bacteroidetes*, *Verrucomicrobia* and *Proteobacteria* became more abundant. High abundances of *Planctomycetes* on *Fucus* sp. biofilms have been described previously in co-occurrence with *Rhodobacterales*, *Gammaproteobacteria*, *Bacteroidetes* and *Verrucomicrobia* [16]. However, these results were not a consistent finding for all examined *Fucus* sp. [16,94]. 

Macroalgae as well as other photosynthetic surface biofilms have been described as communities with at times very high abundances of members of the phylum *Planctomycetes* [10,12,14,15,16,22,25]. Encouraged by these findings and the high abundance of *Planctomycetes*, especially on *Fucus* sp., we examined this phylum on a more resolved level (Figure 1B). It turned out that *Blastopirellula* sp. were dominant in all planctomycetal fractions of the *Laminaria* sp., *Ulva* sp., and *Fucus* sp. biofilms (50.9%, 54.9%, and 76.2%, respectively). Furthermore, the *Ulva* sp. and *Fucus* sp. biofilms were occupied by *Rhodopirellula* sp. (19.4% and 17.8%), *Rubripirellula* sp. (9.7% and 3.8%), and members of the family *Lacipirellulaceae* (12.3% and 0.1%). Instead, the *Laminaria* sp. epiphytic community was not formed by these latter taxa, but comprised yet uncultured members of the family *Pirellulaceae* (38.5%). Other noteworthy taxa of the *Planctomycetes* found in the macroalgae surface microbiome were classified as constituents of the family *Planctomycetaceae*, *Algisphaera* sp. (class *Phycisphaerae*) and the OM190 lineage. The diversity of the particle-associated water fractions of *Fucus* sp. and *Laminaria* sp. was similar to their biofilm composition, whereas *Ulva* sp. adjacent particles contained high amounts of *Rhodopirellula* sp. (69.8%) and *Pirellula* sp. (12.1%), a genus which was only found as a minority in all other samples. A similarity of the planktonic fraction to the biofilm was still found for *Fucus* sp., but the other seawater fractions mainly comprised uncultured *Pirellulaceae*. Several earlier studies focused specifically on planctomycetes found on macroalgae. In accordance with our results, all of the identified taxa could be allocated to the families *Pirellulaceae*, *Lacipirellulaceae*, *Planctomycetaceae* and OM190 lineage [20,21,23]. *Blastopirellula* was found as the dominant genus on *Fucus* sp. as well as *Ulva* sp. sampled at the Portuguese coast, followed by *Rhodopirellula* sp. and other *Pirellulaceae* [19]. These results were validated by our samples from Helgoland Island, emphasizing that epiphytic macroalgal communities seem to be rather independent of their geographic location [9,19,20].

**Figure 2:**
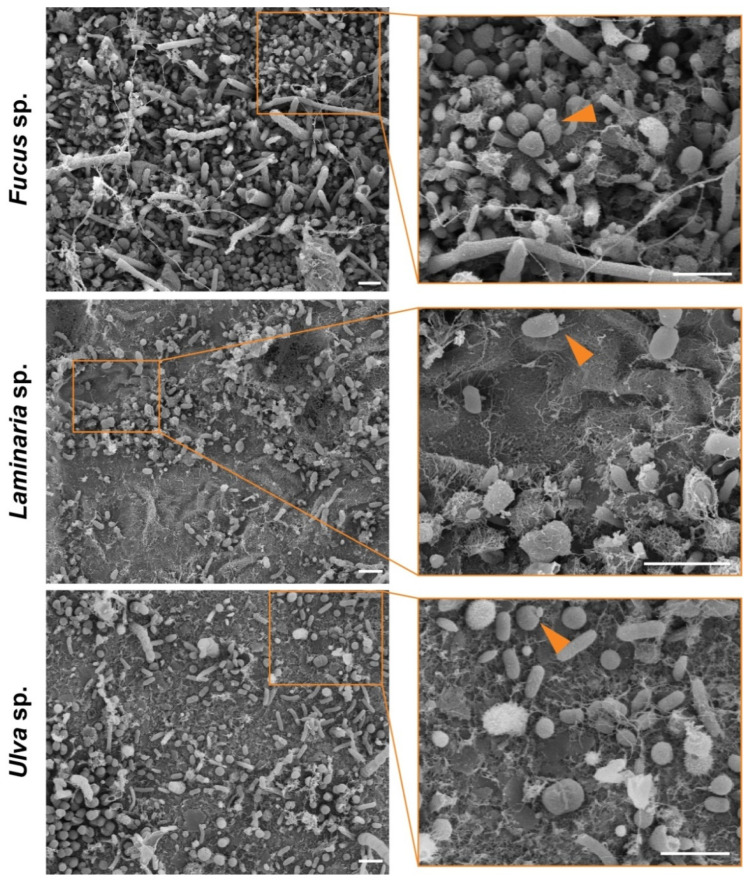
Morphological characteristics of *Fucus* sp., *Laminaria* sp. and *Ulva* sp. biofilms. Overview images (**left**) representatively show the morphological differences between the epiphytic biofilm communities on the surfaces of the different algae. Close-ups (**right**) indicate bacteria which showed budding reproduction by arrowheads, possibly being members of the phylum *Planctomycetes*. Scale bar: 2 µm.

In a second step, the algae samples were analyzed by field emission scanning electron microscopy (FESEM) to determine the different morphologies of the algae-associated surface biofilms. Figure 2 shows the biofilm composition of the sampled *Fucus* sp., *Laminaria* sp., and *Ulva* sp. specimens. *Fucus* biofilms were by far the densest, with less than 5% algal surface visible in all FESEM images (*n* = 16; visual inspection and estimation). While most bacteria appear spherical or rod-shaped with one pole thicker than the other, chains of coccoid cells and tubular-like microorganisms were also visible. In comparison, biofilms of *Laminaria* sp. and *Ulva* sp. were less dense and more non-colonized areas were visible. *Laminaria* sp. biofilms were dominated by rod-shaped bacteria, with about 25–50% of the algal surface lacking microbial epibionts (*n* = 16). Biofilms of the *Ulva* sp. surface showed a very similar degree of colonization as the *Laminaria* sp. samples, while more chain-forming microorganisms were present, and the diversity of bacterial morphologies seemed less broad. A lot of spherical cells with evenly distributed fibers around the whole cell body were visible. While the majority of the microorganisms found on all macroalgae seemed to be dividing by binary fission, budding bacteria could also be spotted on all algal samples (Figure 2, orange arrowheads). However, it is uncertain if these cells belong to members of the class *Planctomycetia* found in all epiphytic communities or if they are members of other budding species. The latter might also be the case since between 1.3% and 1.7% of the found OTUs are derived from non-planctomycetal, but budding bacteria, such as members of the *Hyphomonadaceae*, *Rhodobacteraceae*, or *Alteromonadaceae*. Nevertheless, the abundance of *Planctomycetes* was 0.5–14-fold higher than those of the other budding bacteria, making them an excellent target for further investigation.

### 3.2. Cultivation-Dependent Targeted Isolation of Novel Planctomycetal Strains

The 16S rRNA gene sequence-based analyses and the observation of budding bacteria in the epiphytic communities colonizing the different types of sampled macroalgae both pointed towards the considerable abundance of members of the phylum *Planctomycetes* within the algae-associated biofilms and the surrounding waters. Therefore, we pursued the targeted cultivation of novel planctomycetal strains to obtain axenic cultures of yet uncharacterized taxa.

In initial attempts inoculating media with algae pieces or filters, we mainly obtained fungi, which overgrew the plates within 2–5 days. This observation was made for solid media, liquid enrichment cultures, as well as floating filter assays. To overcome this predicament, we optimized the cultivation conditions by carefully titrating antimicrobial agents, including anti-fungal agents. Together with a pre-treatment of the sample material, fungal growth was eliminated during the initial and subsequent cultivations. 

We also used antibiotics, either carbenicillin or a combination of ampicillin and streptomycin (Supplementary Table S1), against which many known planctomycetes were found to be resistant [44], to inhibit the growth of sensitive bacteria from other bacterial phyla, especially those proliferating considerably faster than planctomycetes. In general, the total number of colonies obtained was higher on peptone/yeast extract-based M1H ASW medium (~80–100 colonies per plate; growth in enrichment cultures after 4–20 days) than on NAG ASW medium (~5–20 colonies per plate; growth in enrichment cultures after 25–90 days), which only contains *N*-acetyl-d-glucosamine as sole carbon and nitrogen source and lacks any complex ingredients. Microscopic analyses showed that M1H ASW medium enriched a broad spectrum of bacteria, even in the presence of carbenicillin (C) or a combination of ampicillin and streptomycin (A/S). Colonies obtained from NAG ASW cultures, however, showed planctomycetal morphology more often and especially cultures containing antibiotic agents were highly enriched in bacteria with planctomycetal morphotypes. 

Especially A/S media were more effective in promoting planctomycetal growth, while carbenicillin tended to reduce the overall colony count. Growth on A/S media was delayed by an average time of ~30 days until first colonies or change of optical density was noted. From the cultivation methods used, biofilm plating, swabbing of algal pieces on solid media, and plating of enrichment cultures from highly selective medium (NAG ASW + A/S) on the corresponding solid medium proved to be the most effective method for selective cultivation of many planctomycetes. Using the floating-filter approach or less selective media, however, fell shorter with respect to the achievement of this goal. 

Colonies of strains with ‘planctomycetal phenotypes’ (pink/red/cream pigmentation and/or budding as cell division mode) were subjected to 16S rRNA gene sequencing. When applying a threshold of <97% 16S rRNA gene sequence identity, four strains unique by the time of the wet-lab work were identified: K22.7^T^ (isolated from *Fucus* sp. biofilm plating), FF011L^T^ (isolated from *Laminaria* sp. floating filter assay), HG15A2^T^ (isolated from *Laminaria* sp. biofilm plating) and EC9^T^ (isolated from an *Ulva* sp. enrichment culture), all of them being members of the recently described order *Pirellulales* [63]. 

### 3.3. Physiological and Chemotaxonomic Characteristics of the Isolates

**Figure 3:**
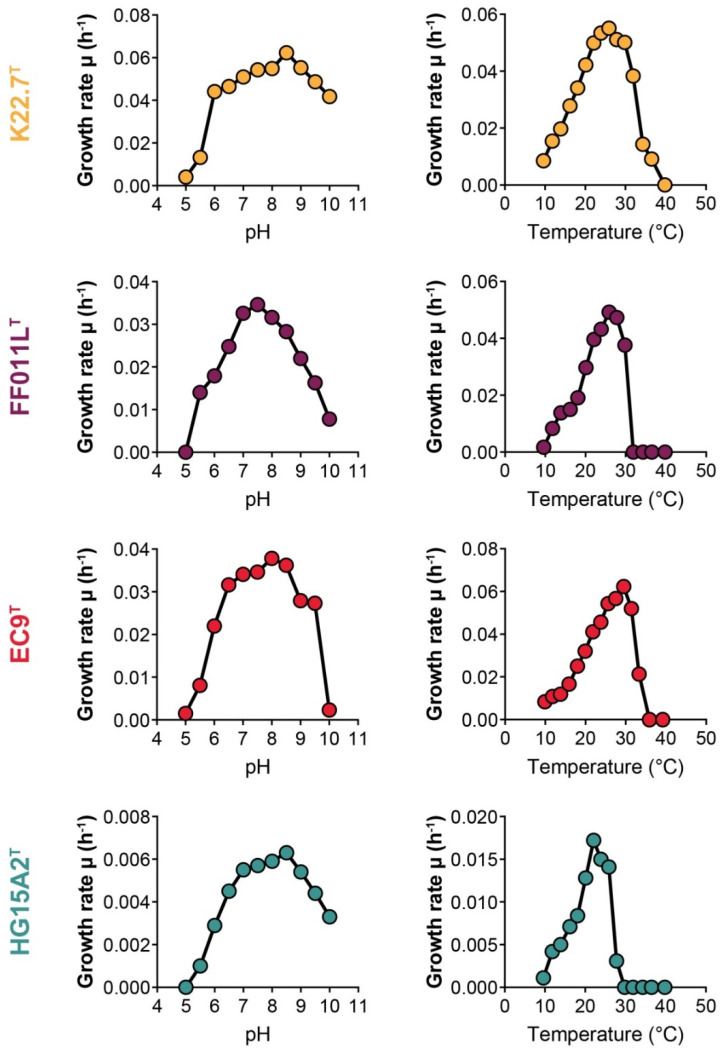
Temperature and pH optima of strains K22.7^T^, FF011L^T^, EC9^T^ and HG15A2^T^. Growth was determined by optical density measurements at 600 nm and growth rates were calculated for cultures during the exponential phase. Each dot represents the mean of duplicate measurements.

Starting from the axenic cultures obtained for strains K22.7^T^, FF011L^T^, HG15A2^T^, and EC9^T^, we conducted additional experiments in order to characterize important physiological and chemotaxonomic characteristics. During cultivation of the strains, all four turned out to be chemoorganoheterotrophs capable of using *N*-acetyl-d-glucosamine as sole carbon and nitrogen source, but with a preference for a more complex medium also containing peptone, yeast extract, and glucose. The strains displayed a mesophilic growth profile (Figure 3, Table 1), with a tolerated temperature range starting at 10 °C and rising to temperatures from 26 °C (strain HG15A2^T^) to 37 °C (strain K22.7^T^). The optimal growth temperatures (T_opt_) fall between 22 °C (strain HG15A2^T^) and 30 °C (strain EC9^T^). The maximal growth rates at T_opt_ (Table 1) in M1H NAG ASW medium were calculated to be between 0.018 and 0.062 h^−1^ which corresponds to doubling times between approximately 11 h (strain EC9^T^) and 39 h (strain HG15A2^T^). We observed a clear correlation of higher T_opt_ and higher growth rates between the analyzed strains.

The pH tolerance was broad for all strains, spanning a range from pH 5.0 or 5.5 to pH 10.0 (Figure 3, Table 1). The optima for growth were pH 7.5 for strains FF011L^T^ and EC9^T^ and pH 8.5 for strains K22.7^T^ and HG15A2^T^ (Table 1), making all strains neutrophiles. However, when evaluating the results of the pH tolerance tests, we found that the strains modified the pH values within the buffered medium towards values between pH 7.0 and 8.0 which rather suggests a pH optimum at ~7.5. Usually, alterations of pH levels during bacterial growth are a known phenomenon caused by the release of acidic or basic compounds as metabolic end products [95]. In cases where pH values should not be adjusted by the culture, buffer concentration higher than the 10 mM used here would be necessary.

All strains tested positive for cytochrome oxidase activity. The same was true for the determination of catalase activity. Fatty acid analyses revealed C_18:1_ω9c as the major compound in strains K22.7^T^, FF011L^T^, and HG15A2^T^, making up 42.5%, 55.1%, and 34.8% of detected fatty acids, respectively, while C_16:0_ was the second most abundant fatty acid. In strain EC9^T^, it was the other way around, the major fatty acid component being C_16:0_ with 37.3%. A detailed overview of fatty acid contents of strains described in this study and related planctomycetal type strains is provided in Supplementary Table S4. 

**Figure 4:**
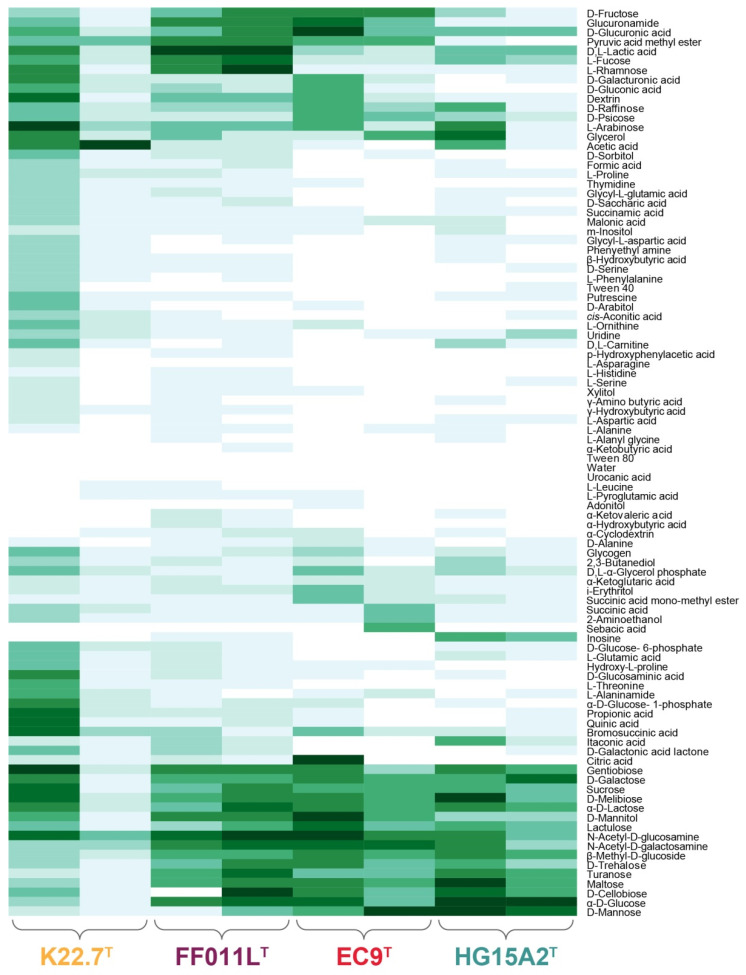
Heatmap of substrate utilization patterns of strains K22.7^T^, FF011L^T^, EC9^T^ and HG15A2^T^. Two biological replicates were performed using the MicroLog GN2 substrate plates. Color scale shows substrate usage in percent utilization (white: 0%, dark green: 100%).

Substrate utilization was determined with the MicroLog 96 substrate spectrum plates for Gram-negative bacteria. Two replicate plates were analyzed for each strain, and data were visualized as a heatmap dendrogram (Figure 4). However, analysis of the substrate spectra raised the question of the replicability of results. While the qualitative statement concerning whether a substrate could in principle be used by a strain is feasible, an inference of the quantitative ‘strength’ of utilization was not possible (Figure 4). This problem has been described before for bacteria of other phyla [96], but also for *Planctomycetes* [97]. A possible explanation for the deviant results might be the use of different cultures for inoculation of the assays. It is possible that these cultures were at different developmental stages, even though inoculated from the same pre-culture, thereby featuring a different ratio of planktonic and attached-living cells that could likely influence the substrate preferences of the culture [98].

We found that strains FF011L^T^ and K22.7^T^ were able to utilize 89 and 88 of all 95 tested substrates, respectively, while strains HG15A2^T^ and EC9^T^ only were capable to consume 75 and 59 substrates, respectively (Figure 4). The substrate spectra did not show any correlation with the genome size or growth rate. Interestingly, and in contrast to the two other strains, EC9^T^ and HG15A2^T^ failed to utilize most of the tested amino acids. Additionally, strain EC9^T^ was less prone to the utilization of various carboxylic acids. In total, 34 out of 95 substrates were used by all strains in all replicates: 2-aminoethanol, bromosuccinic acid, d-fructose, d-galactose, d-glucuronic acid, d-mannitol, d-mannose, d-melibiose, d-psicose, d-raffinose, d-trehalose, d,l-lactic acid, d,l-α-glycerol phosphate, dextrin, gentiobiose, glucuronamide, glycerol, glycogen, i-erythritol, l-arabinose, l-fucose, l-rhamnose, lactulose, maltose, mono-methyl-succinate, *N*-acetyl-d-galactos amine, *N*-acetyl-d-glucosamine, succinic acid, sucrose, turanose, α-d-glucose, α-d-lactose, α-ketoglutaric acid, and β-methyl-d-glucoside. The only substrates not used by any strain were Tween 80 and urocanic acid. As expected, this range of substrates being accepted by all strains includes d-glucose and *N*-acetyl-d-glucosamine, the latter being frequently used to selectively enrich planctomycetes from environmental samples [43]. Additionally, all four strains were able to utilize d-mannitol, a sugar alcohol frequently found in the cell walls of brown algae, especially *Laminaria* species, in which it can account for up to 25% of the dry weight in algal fronds [99]. The strains were also capable of metabolizing sugars such as glucose, mannose, galactose, and arabinose, which are monomers present in hydrolysates of brown macroalgae, like *Laminaria* and *Fucus*, and in species of the green seaweed *Ulva* [100,101]. Other frequent algal sugars like fucose and rhamnose are also consumed by all strains, implying that planctomycetes can feed on algal material, maybe even upon active degradation [25].

**Figure 5:**
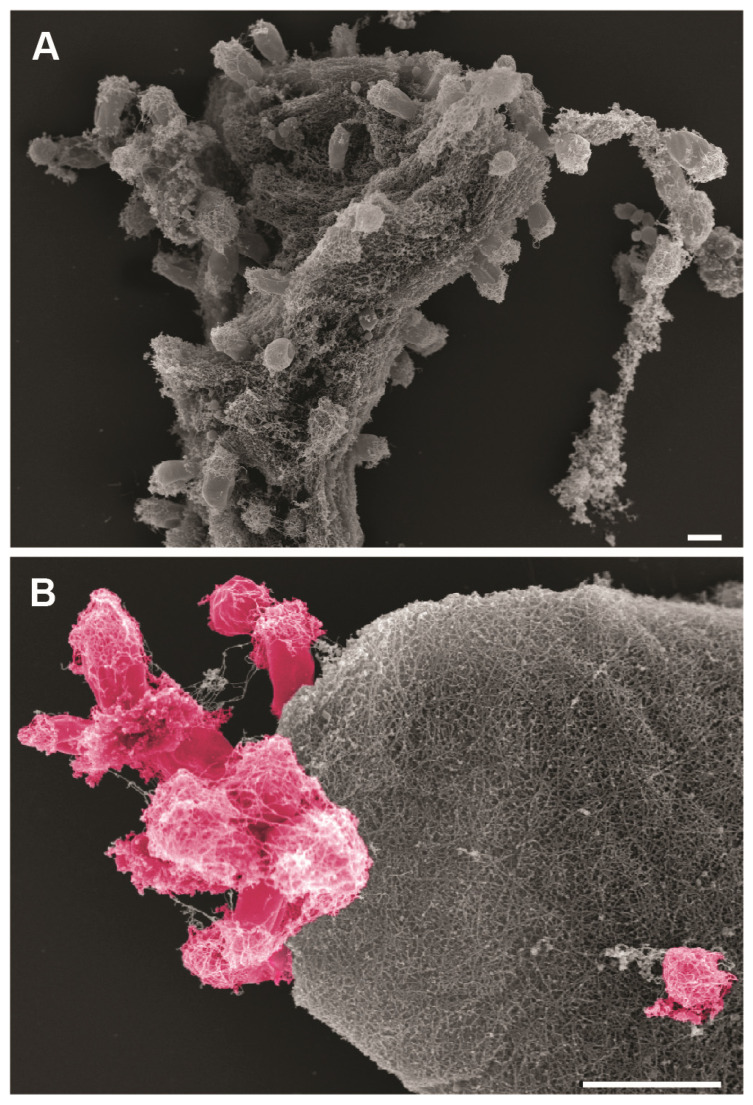
Attachment of strain K22.7^T^ to algal particles. Strain K22.7^T^ was cultivated in AMS medium containing *Fucus* sp. particles as sole carbon and nitrogen source. Attachment of K22.7^T^ cells to algal particles was visualized by SEM (**A**,**B**) and the false color image (**B**) shows bacterial cells (hot pink) attached to an algal particle in higher magnification. No budding cells were observed in the culture at day 21 of incubation, but pili-like fibers covered the pole caps of individual cells. Scale bars 1 µm.

To further investigate if the novel strains were indeed able to grow on algal particles, we determined the fate of strain K22.7^T^ when providing algal powder of a *Fucus* species as sole carbon and nitrogen source. The combination of the strain and the powder was chosen on purpose as strain K22.7^T^ was originally isolated from a *Fucus* biofilm. Growth of the strain on the algal particles was documented by scanning electron microscopy (SEM) (Figure 5), which shows a tight attachment of the planctomycetal cells to the particles, consistent with their suspected capability to feed on algal matter. The ability of *Planctomycetes* to degrade complex polysaccharides, including chondroitin sulfate and laminarin, has been previously reported [97] and species of the genus *Rhodopirellula* were found to encode more than 100 sulfatases in their genomes, possibly enabling the degradation of a variety of different substrates of high chemical complexity [38]. This finding is especially interesting in light of the above mentioned hypothesis of planctomycetes being active degraders of their hosts [25].

### 3.4. Morphology and Cell Biology

**Figure 6:**
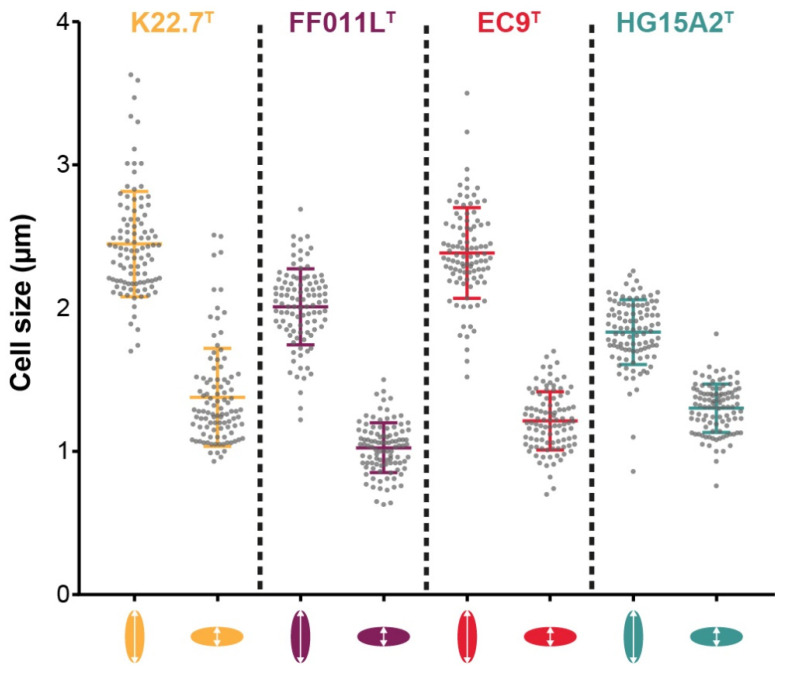
Cell sizes of the isolated planctomycetal strains K22.7^T^, FF011L^T^, EC9^T^ and HG15A2^T^. Average cell size of all strains was investigated by light microscopy and under phase-contrast illumination. Cell size was determined by measuring 100 individual cells per strain. Each dot represents one measurement and median and standard deviation values are indicated.

**Figure 7:**
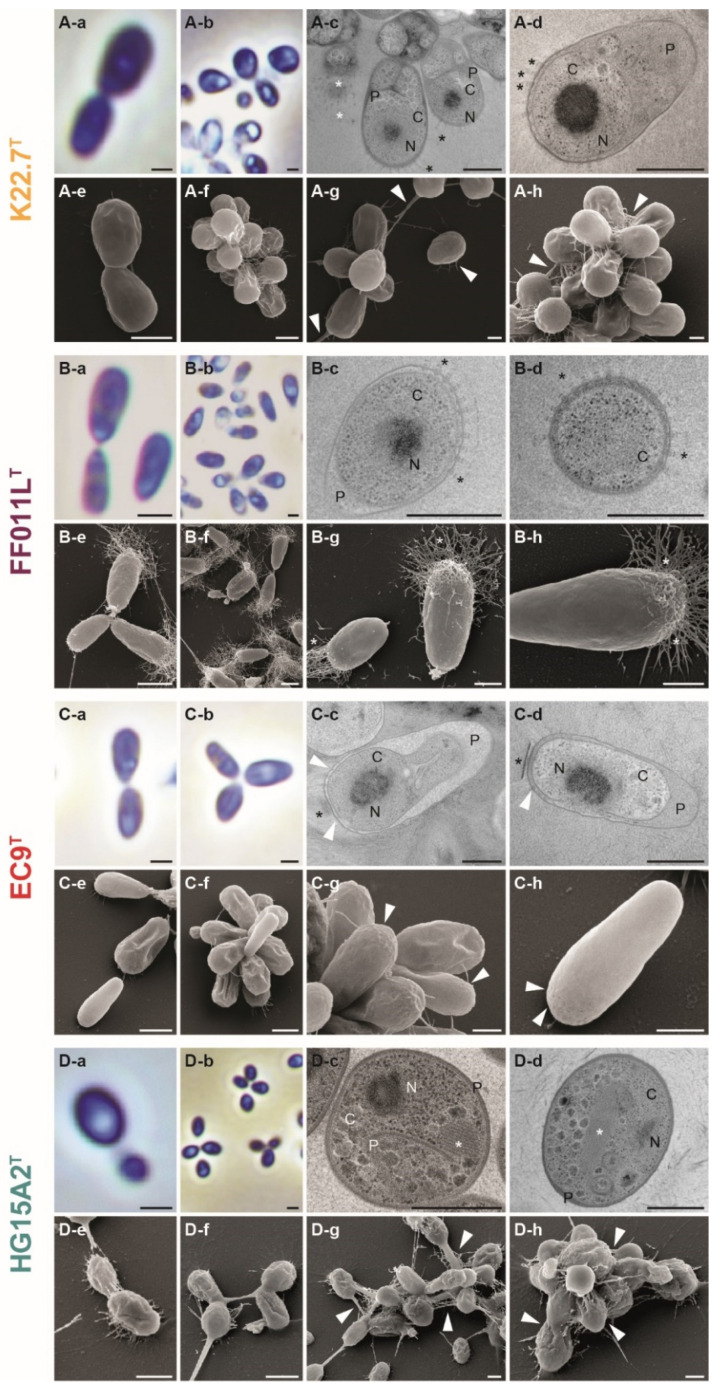
Morphology of strains K22.7^T^ (**A**), FF011L^T^ (**B**), EC9^T^ (**C**) and HG15A2^T^ (**D**). Phase contrast light microscopy (**a**,**b**), transmission electron microscopy of thin sections (**c**,**d**) and scanning electron microscopy (**e**–**h**) of all strains. (C) Cytoplasm; (N) condensed nucleoid; (P) periplasm. Explanations to asterisks and arrowheads are given in the text. Scale bars are 1 µm (**a**–**c**,**f**) and 500 nm (**c**,**d**,**g**,**h**).

For investigation of the cellular morphology and cell size of strains K22.7^T^, FF011L^T^, EC9^T^, and HG15A2^T^ (Figure 6), we performed light, scanning, and transmission electron microscopy (Figure 7). Strain K22.7^T^ was 2.5 ± 0.4 µm in length and 1.4 ± 0.3 µm in width. Strain FF011L^T^ was smaller, measuring 2.0 ± 0.3 µm in length and 1.0 ± 0.2 µm in width. With its elongated cell bodies, cells of strain EC9^T^ measured 2.4 ± 0.3 µm in length and 1.2 ± 0.2 µm in width. The smallest of the novel strains was HG15A2^T^, measuring 1.8 ± 0.2 in length and 1.3 ± 0.2 µm in width (Figure 6, Table 1). This smallest of the four strains showed an egg-shaped morphology. It divides by polar budding (Figure 7D). Cells of strain HG15A2^T^ occurred as single cells or aggregates of 3–10 cells (Figure 7D-g,h). 

Several planctomycetal strains show a dimorphic lifecycle involving sessile mother cells and flagellated daughter cells. Attachment or aggregate formation in liquid cultures often leads to a higher viscosity when cultures reached the stationary phase, while high mobility of smaller swimmer cells turned out to be a characteristic feature during the exponential phase. SEM imaging revealed the existence of a thick extracellular matrix compound, in which the cells were embedded. This compound also interconnected single cells through thick appendages (up to 500 nm) that could be very short but could also reach several micrometers in length (Figure 7D-e–h). In line with the higher viscosity of stationary cultures, the connective extracellular compound was present in higher quantities in late stationary phase cultures in comparison to exponential cultures. Transmission electron microscopy (TEM) of thin sections revealed a double membrane system, compartmentalizing the cells in a *Planctomycetes*-typical periplasmic space with several invaginations and a cytoplasmic space which contained the condensed nucleoid (Figure 7D-c,d). In addition, some cells showed intracytoplasmic parallel-running membrane stacks with individual lengths from 300 nm to 1.5 µm (Figure 7D-c,d, white asterisks). Similar structures were detected in other planctomycetal species before, but no specific role or function was suggested [102,103]. The anammox (anaerobic ammonia oxidation) planctomycete *Candidatus* Kuenenia stuttgartiensis was described to harbor these tubule-like structures in its supplementary compartment, the anammoxosome [104]. 

The observed microstructures were hypothesized to be related to cytoskeletal elements or displaying a higher organization of an abundant protein which assembles into the observed meta-structure [104,105]. A recent study indeed suggested that these stacked microstructures are indeed a higher organization of an abundant protein as they specifically co-localized with antibodies against a nitrite oxidoreductase (NXR) [106]. The function of the microstructures in aerobic planctomycetes however remains elusive.

Phase contrast imaging revealed a typical planctomycetal cell shape for strain K22.7^T^, for which cells are slightly elongated with a pear-like morphology (Figure 7A). Cells reproduce by polar budding (Figure 7A-a). No motility or chain formation were observed (Figure 7A-b). SEM imaging of cells grown in standard M1H NAG ASW medium displayed a smooth cell surface with no crateriform structures visible (Figure 7A-e), indicating the embedding in a dense extracellular matrix. Cells form aggregates and small rosettes consisting of 3–6 cells and usually connected at the narrower pole (Figure 7A-a,d) by the release of sparse fibers from the matrix (Figure 7A-g,h, white arrowheads). The extracellular compound was also visible in liquid cultures, in which cells formed dense biofilms on glass surfaces when incubated under constant agitation. Notably, the strain showed a different morphology when cultured with algal powder as carbon and nitrogen source (Figure 5B,C).

Cells of strain FF011L^T^ had a pear-like cell shape with one pole wider than the other (Figure 7B) and the cells divide by polar budding (Figure 7B-a). No aggregate formation in liquid culture (Figure 7B-b) and no motile cells were observed during phase contrast microscopy. SEM imaging revealed a network of fiber-like structures at the wider cell pole of strain FF011L^T^ (Figure 7B-e–h) and the existence of a holdfast structure at the narrower pole (Figure 7B-e,g). The two features allowed the cells to connect closely via the narrower pole, while the fiber-like structures assembled the cells into loose aggregates, thereby allowing the formation of rosettes of up to 10 cells. The tight mesh of fibers was not only present in large, mature cells, but also in smaller cells (Figure 7B-g). TEM imaging of thin sections of strain FF011L^T^ showed cells with a condensed nucleoid and often enlarged periplasmic space (Figure 7B-c,d). The rim of the wider pole of the cells was seamed with tubule- or fiber-like structures, which stretch through the outer most membrane into the periplasmic space (Figure 7B-c,d, black asterisks). The top-down visible in Figure 7B-d indicated that the structures are distributed evenly around the cap of the wider cell pole. 

Strain EC9^T^ displayed a distinctly slim pear-shaped morphology with one pole being wider than the other (Figure 7C). Cells formed large aggregates and rosettes with large and small cells attached to each other at the narrower pole (Figure 7C-a,b,f). Cells divide by polar budding (Figure 7C-f). Motility was not observed at the analyzed time points. TEM imaging showed cells with a condensed nucleoid as well as a partially invaginated periplasmic space and a cytoplasmic space of varying size parted by two double membranes (Figure 7C-c,d). In addition, many cells showed an extracellularly attached, stacked structure which seemed to be tangential to the wider cell pole (Figure 7C-c,d, black asterisk). However, no similar structure could be observed in SEM images. Furthermore, cells of strain EC9^T^ showed crateriform structures, evenly distributed at the cap of the wider cell pole (Figure 7C-g,h, white arrowheads) that were also visible in cells investigated by TEM imaging (Figure 7C-c,d, white arrowheads). Not unlike strain FF011L^T^, dense fiber-like appendages were formed on the broad pole, while the narrower pole mediated the attachment to the algal particles.

For strain K22.7^T^, TEM images revealed a similar cellular structure as strains EC9^T^ and FF011L^T^, showing a condensed nucleoid, partially enlarged periplasm and a cytoplasm with ribosomes (Figure 7A-c,d). The strain also displayed tubule- or fiber-like structures which locate through the outermost membrane into the periplasmic space (Figure 7A-c,d, black asterisks) seen before in strain FF011L^T^. These structures potentially correspond to the fibers observed by SEM in standard medium for strain FF011L^T^ and algal medium for strain K22.7^T^ as they are also located at the wider cell pole. Similar structures were previously described in related planctomycetes [102], but their organization or function was not further investigated. However, the elucidation of the architecture of type IV pili in *Thermus thermophilus* showed that the pili apparatus possesses structural elements very similar to those seen in our strains K22.7^T^ and FF011L^T^ [107]. Likewise, the localization of the type IV pilus was identical, spanning through the periplasmic space to the outside of the cell. While type IV pili are associated with a wide range of functions, such as twitching motility, DNA uptake and microcolony formation [108], another role has been recently suggested for similar structures observed on the surface of the distant relative *Planctopirus limnophila*, a member of the order *Planctomycetales* [41]. It has been observed that the appendages seem to protrude from the crateriform structures and that they are capable to bind complex branched carbohydrates, implying a role of the appendages in nutrient uptake [41]. The formation of appendages by strain K22.7^T^ in an algal medium rich in suspended macromolecules, but a lack of their formation when fewer complex sugars are provided, is consistent with this hypothesis [41,109].

### 3.5. Phylogenetic Inference and Analysis of Genome-Encoded Features

**Figure 8:**
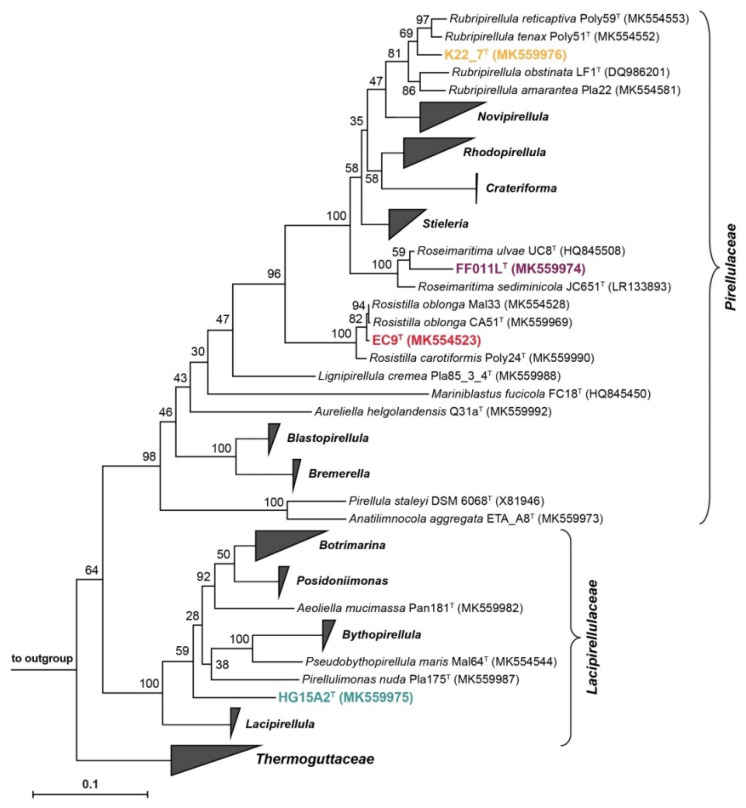
Phylogeny of the novel strains within the order *Pirellulales*. For the 16S rRNA analysis, maximum likelihood estimation-gained bootstrap values after 1000 re-samplings are given at the nodes in percent. The outgroup consists of three 16S rRNA genes from the family *Planctomycetales*.

The construction of a genome-based tree from a multilocus sequence analysis (MLSA) as well as a 16S rRNA gene sequence-based phylogenetic tree confirmed the placement of the four novel strains in the order *Pirellulales* (see Section 3.6, Section 3.7, Section 3.8 and Section 3.9, Figure 8 and Supplementary Figure S1). In this order, strains K22.7^T^, EC9^T^ and FF011L^T^ appeared to belong to different genera of the family *Pirellulaceae*, whereas strain HG15A2^T^ was identified as member of the family *Lacipirellulaceae*. 

### 3.6. Strain K22.7^T^ and the Genus Rubripirellula

**Figure 9:**
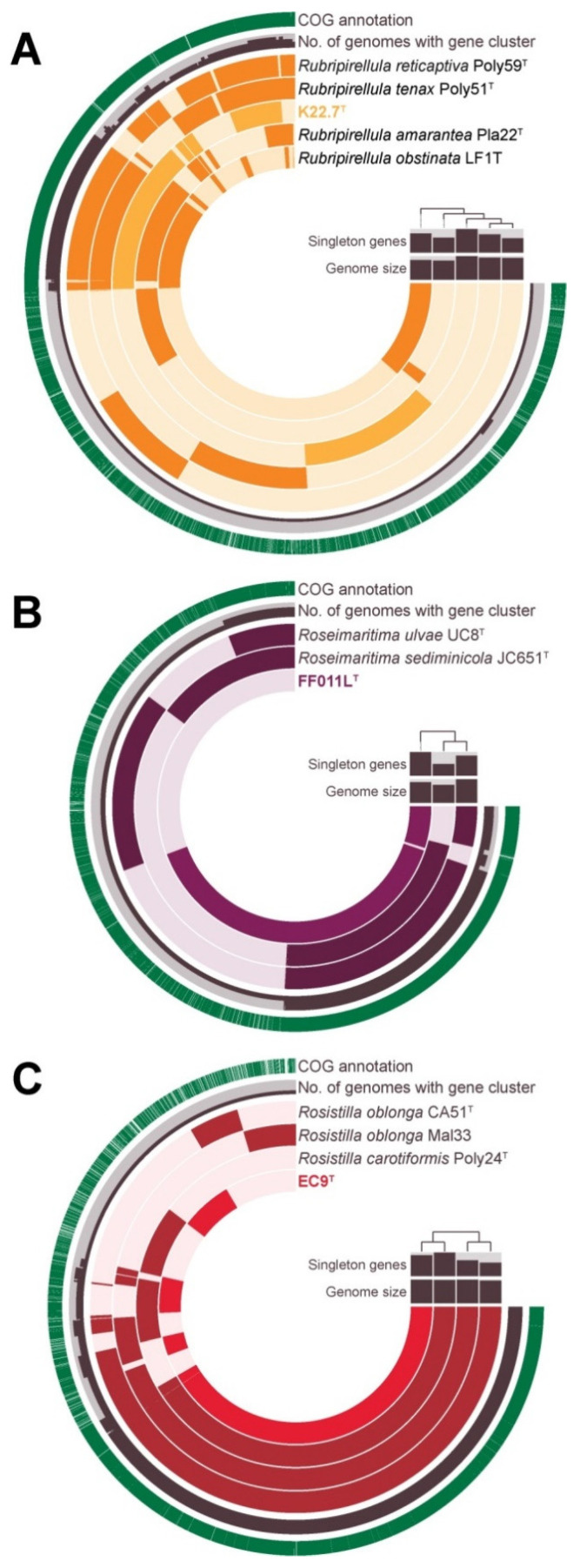
Pan genome of the novel strains in the context of their genera. Each open circle represents the pan genome of all strains but is colored darker when the gene is present in the respective genome. The trees reflect on the relatedness of the strains based on the absence/presence of genes. The outer circles show the number of genomes in which a gene is present and if it has a COG annotation (dark grey) thereby relaying information on how good the genes are annotatable. (**A**): Strain K22.7^T^ and the genus *Rubripirellula.* (**B**): Strain FF011L^T^ and the genus *Roseimaritima*. (**C**): Strain EC9^T^ and the genus *Rosistilla*. Strain HG15A2^T^ was omitted since it is currently the sole member of the novel genus *Adhaeretor*.

In both phylogenetic trees (Figure 8 and Supplementary Figure S1), strain K22.7^T^ clustered within the genus *Rubripirellula* [29,61]. The assumption that strain K22.7^T^ belongs to this genus was confirmed by average amino identities (AAI) and the percentage of conserved proteins (POCP) given that all values were distinctly above the proposed genus thresholds of 60–80% [110] and 50% [84], respectively (Supplementary Figure S2). Average nucleotide identities (ANI) distinctly below the species threshold of 95% [111] in turn suggest that strain K22.7^T^ does not belong to one of the previously described strains of the genus. This notion is supported by *rpoB* sequence identity values below the proposed species threshold of 95.5% [80] and above the newly defined genus threshold of 75.5–78.0% [61]. Eventually, 16S rRNA gene sequence identities between 96.0% and 97.2% also suggest the classification of strain K22.7^T^ as a representative of a new species within the genus *Rubripirellula* as these values fall below the species threshold of 98.7%, but above the genus threshold of 94.5% (Supplementary Figure S2) [112]. The genome sizes for the described strains within the genus *Rubripirellula* vary between 6.95 and 8.54 Mb with strain K22.7^T^ having the largest genome and the only one being completely closed (Supplementary Table S5). With 57.3%, the strain also has the highest DNA G+C content of the genus *Rubripirellula*. The strain features the most genes with a size >15 kb, the so-called giant genes, and the highest number of transposable elements. In contrast to the other members of the genus, strain K22.7^T^ harbors two 16S rRNA gene copies and two 23S/5S rRNA operons. The strain has the fewest tRNAs. Due to its large genome, strain K22.7^T^ harbors the highest number of 6512 total genes, of which 6461 are protein-coding. The strain also has the highest count of 4320 unspecified hypothetical proteins. While the five strains of the current genus share a core genome of 1966 genes with a high density of annotated genes, 2540 of strain K22.7^T^’s genes were not found in any of the other genus members (Figure 9A).

### 3.7. Strain FF011L^T^ and the Genus Roseimaritima

Upon inspection of the 16S rRNA gene sequence- and the MLSA-based phylogenetic trees (Figure 8 and Supplementary Figure S3), it became evident that strain FF011L^T^ clusters within the genus *Roseimaritima* [29,65]. Its phylogenetic position including assignment to the genus *Roseimaritima* is supported by both, the POCP as well as the AAI values, found to be above the respective genus thresholds of 50% and 60–80%, respectively [84,110] (Supplementary Figure S3). It was however not clear from the trees which one of the two already described strains of the genus is the current closest relative of the novel strain and whether strain FF011L^T^ also constitutes a novel species. The ANI value distinctly hinted to the interpretation as novel species, a finding that is additionally supported by the *rpoB* gene sequence identity (Supplementary Figure S3) and the 16 rRNA gene sequence identity (Supplementary Figure S3) as all values for these two parameters were above the genus, but below the species threshold. This said, strain FF011L^T^ can be clearly delineated from the two described species in the genus and should thus be regarded as the member of a novel species within the genus.

In direct comparison of strain FF011L^T^ and the two already described species of the genus *Roseimaritima*, *R. ulvae*, and *R. sediminicola*, the strains’ genome sizes fell between 6.25 and 8.21 Mb (Supplementary Table S6). The genomes cover a DNA G+C content range from 54.5 to 62.4%, with strain FF011L^T^ having the lowest G+C content. Automated gene annotation of the genome of strain FF011L^T^ yielded 5533 predicted genes, of which 5482 are annotated as proteins. While these values put the strain between its two described relatives, strain FF011L^T^ encodes these features with the highest count per Mb. The three genomes share 2507 genes, whereas strain FF011L^T^ also harbors 2300 genes that are not shared within the genus (Figure 9B). Strain FF011L^T^ does not have many giant genes, but 41 transposable elements. As the only member of its genus, strain FF011L^T^ features two 16S rRNA genes and two 23S/5S rRNA operons, but has the fewest tRNA genes.

### 3.8. Strain EC9^T^ and the Genus Rosistilla

When reviewing 16S rRNA gene sequence- as well as MLSA-based phylogeny of strain EC9^T^ and its close neighbors (Figure 8 and Supplementary Figure S1), it became apparent that the novel strain belongs to the only recently described genus *Rosistilla,* which comprises *R. oblonga* and *R. carotiformis* [31]. The assessment that these species and strain EC9^T^ belong to the same genus is confirmed by AAI and POCP values distinctly above the genus thresholds (Supplementary Figure S4). With an ANI value <90%, an *rpoB* gene identity of 92.3%, and a 16S rRNA gene sequence identity of 98.6% (Figure 9C and Supplementary Figure S4), it is also conclusive that strain EC9^T^ does not belong to the species *Rosistilla carotiformis*. The identity values between strain EC9^T^ and the *Rosistilla oblonga* type strain CA51^T^ are however less clear: while the ANI value of 90.3% is clearly below the accepted species threshold of 95% [111], the *rpoB* gene identity of 97.3% and the 16S rRNA gene sequence identity of 99.6% both fall above the proposed species thresholds of 95.5% and 98.7%, respectively [80,112]. In case of the phylum *Planctomycetes*, it has been shown before that strains often belong to different species according to ANI analysis despite 16S rRNA sequence identities >99% [36,109,113]. We therefore assigned strain EC9^T^ to a separate species based on the ANI value being distinctly below the species threshold, especially as this result was supported by the evaluation of the *Rosistilla* genomes: while the genomes of the *Rosistilla oblonga* strains have a very similar DNA G+C content of 58.1% and 58.2%, strain EC9^T^ has a lower DNA G+C content of 57.9%. The genomes reach a size between 7.25 and 7.51 Mb (Supplementary Table S7), thereby carrying 5298 to 5576 predicted genes, 4012 of which are shared between all four strains (Figure 9C). The two *Rosistilla oblonga* strains CA51^T^ and Mal33 exclusively shared 256 further genes, while EC9^T^ and CA51^T^ only shared 58, EC9^T^ and Mal33 shared 57, and EC9^T^, Mal33, and CA51^T^ shared 155 genes. Strain EC9^T^ and *Rosistilla carotiformis* Poly24^T^, however, shared 264 genes, thereby indicating a genetic distance between strain EC9^T^ and the species *Rosistilla oblonga*.

### 3.9. Strain HG15A2^T^ and the Family Lacipirellulaceae

Both, MLSA- as well as 16S rRNA sequence-based phylogeny (Figure 8 and Supplementary S1) suggested that strain HG15A2^T^ is a member of the only recently proposed and then expanded planctomycetal family *Lacipirellulaceae* [42,63,114]. The placement as a new genus is supported by AAI and POCP values found to fall below (in a single case slightly above) the genus thresholds of 60–80% and 50%, respectively [84,110] (Supplementary Figure S5). In addition, the determined *rpoB* gene identity values are in line with our conclusion to delineate the strain from the known genera in the family *Lacipirellulaceae*. All 16S rRNA gene sequence identities are below the genus threshold of 94.5%, but above the family threshold of 86.5% [112]. Within the *Lacipirellulaceae*, the genome of strain HG15A2^T^ is a rather typical representative member of this taxon (Supplementary Table S8). 

### 3.10. Genome-Based Analysis of Metabolic Features of the Novel Isolates

As all four novel isolates are aerobic heterotrophs, we expected to find genes encoding a canonical primary carbon metabolism. Nevertheless, since the central carbon metabolism of the class *Planctomycetia* has not been studied in detail, we analyzed the genomes of the isolates for the presence of genes encoding enzymes involved in glycolytic pathways and gluconeogenesis, the tricarboxylic acid (TCA) cycle and anaplerotic reactions such as the phosphoenolpyruvate-pyruvate-oxaloacetate node and the glyoxylate shunt. Although genes coding for most of the enzymes required for the activity of central metabolic pathways were identified, there were also some anomalies that might require additional attention in the future.

Strain K22.7^T^ harbors the entire set of genes coding for enzymes of glycolytic pathways. Next to the common Embden–Meyerhof–Parnas pathway, the alternative Entner-Doudoroff pathway as well as the pentose phosphate pathway appear to be functional. Strain K22.7^T^ is currently the sole member of the genus *Rubripirellula* harboring the gene for a 2,3-bisphosphoglycerate-dependent phosphoglycerate mutase (encoded by *gpmA*) in addition to the 2,3-bisphosphoglycerate-independent isoenzyme (encoded by *gpmI*) found in all current members of the genus. The strain encodes all enzymes of the TCA cycle, but lacks the glyoxylate shunt. The latter appears to be a common feature of members of the class *Planctomycetia* when considering that genes for the two involved enzymes, isocitrate lyase and malate synthase, have not been identified in any isolate belonging to this class [31]. Furthermore, all characterized strains of the genus *Rubripirellula* and strain K22.7^T^ probably lack the enzymatic activity required to convert fructose-1,6-bisphosphate into fructose-6-phosphate during gluconeogenesis. This would imply that none of these strains is capable of *de novo* biosynthesis of monosaccharides, which in turn are essential for the biosynthesis of cell wall components, nucleic acids, and certain amino acids.

In the genome of strain EC9^T^ and its close relatives, the genes for all enzymes involved in the central carbon metabolism are present, except for the two above-mentioned enzymes of the glyoxylate shunt [31].

For strain FF011L^T^, the encoded genes suggest intact glycolytic pathways and a complete TCA cycle. The analysis also points towards an incomplete set of gluconeogenesis enzymes and the lack of the *sucA* gene coding for the E1 component of the 2-oxoglutarate dehydrogenase complex in several members of the family including strain HG15A2^T^. Nevertheless, the four novel isolates appear to have a central carbon metabolism similar to that of other heterotrophic bacteria.

Members of the phylum *Planctomycetes* have been suggested to be a promising source for the isolation of novel secondary metabolites with important bioactivities, e.g., anti-oxidative or antimicrobial properties [39,40,97,115]. To address this potential, we identified and analyzed biosynthetic gene clusters (BGCs) encoded by the novel isolates that might be of importance in that regard. The analysis yielded 4–7 putative BGCs relevant for secondary metabolite production in the four isolates and a correlation between genome size and the number of BCGs was found to some extent (Supplementary Table S9). The identified BGCs can be split into two groups: (I) clusters coding for enzymes involved in isoprenoid and terpenoid production and (II) clusters containing larger genes coding for modular multi-domain proteins belonging to the classes of polyketide synthases (PKSs) or non-ribosomal peptide synthetases (NRPSs). Clusters falling in group I encode early biosynthetic proteins of carotenoid- or hopanoid-forming pathways, e.g., phytoene and squalene synthases or polyprenyl synthetases and terpene cyclases [116,117]. The presence of such genes is in line with the presence of carotenoids in three out of the four isolates that show a pink to red pigmentation or the presence of hopanoids, which have also been found to be produced by planctomycetes [118,119]. Products obtained from PKS and NRPS proteins encoded in clusters of group II have a broad range of functions, e.g., as antibiotics, surfactants, secondary lipids, siderophores, etc. [120,121,122]. Strains K22.7^T^, FF011L^T^, and EC9^T^ harbour four such clusters belonging to group II, whereas only two group II BCGs could be identified in strain HG15A2^T^, which has a considerably smaller genome compared to the other three strains. 

The predicted BGCs only showed low similarity of <10% to hits of the most recent version of antiSMASH. This indicates that the secondary metabolites produced by proteins encoded in these cluster, although having a similar function as known compounds, are likely structurally different from previously characterized molecules identified in bacteria and fungi [39].

### 3.11. Conclusions

As planctomycetes are known to often occur attached to algal surfaces, the aim of this study was to investigate the composition of the bacterial epiphytic community on different macroalgae. Despite the long generation times of *Planctomycetes* compared to other heterotrophic bacteria inhabiting such surfaces [94], we found that the phylum constitutes the dominant fraction of bacteria on *Fucus* sp. biofilms and also occurs on *Ulva* sp. and *Laminaria* sp. that were sampled in the North Sea. In order to gain more insight into the mechanisms by which those slow-growing bacteria colonize macroalgae in a competitive environment, four novel planctomycetal strains belonging to the order *Pirellulales* were brought into axenic culture and it was exemplarily shown for strain K22.7^T^ that such strains have the ability to grow on algal particles. Further experiments, including setups with living algal material, will be needed to investigate the true nature of planctomycetal biofilm formers and to solve the question whether their relationship with algae is mutually beneficial, opportunistic or simply parasitic.

Based on the results of our polyphasic analysis of four novel planctomycetal isolates that included chemotaxonomy, physiological features, morphology, cell biology, phylogenetic markers, and genome comparisons, we conclude that strain K22.7^T^ belongs to a novel species in the genus *Rubripirellula*, for which we propose the name *Rubripirellula lacrimiformis*. Furthermore, strain FF011L^T^ constitutes a novel species of the genus *Roseimaritima* with the proposed name *Roseimaritima multifibrata* and strain EC9^T^ is a member of a yet uncharacterized species of the genus *Roseimaritima*, for which we introduce the name *Rosistilla ulvae*. Finally, strain HG15A2^T^ constitutes a novel species of a novel genus within the family *Lacipirellulaceae*, for which the name *Adhaeretor mobilis* is proposed.

## 4. Genus and Species Protologues

### 4.1. Description of Adhaeretor gen. nov.

*Adhaeretor* (Ad.hae.re’tor. N.L. masc. n. *Adhaeretor* from L. v. *adhaerere* to stick, to cling; an adhering bacterium).

Cells are round rice grain-shaped to round and form multicellular rosettes and aggregates. Cells divide by polar budding, are motile during the exponential growth phase and do not form spores. Extracellular matrix and biofilm formation in liquid culture are observed during cultivation. Aerobic, mesophilic, and neutrophilic growth properties. The molar DNA G+C content is 55–56%. The predominant cellular fatty acid of the type species is oleic acid (C_18:1_ ω9c). The genus belongs to the phylum *Planctomycetes*, class *Planctomycetia*, order *Pirellulales*, family *Lacipirellulaceae*. The type species of the genus is *Adhaeretor mobilis*.

### 4.2. Description of Adhaeretor mobilis sp. nov.

*Adhaeretor mobilis* (mo’bi.lis. L. masc. adj. *mobilis* movable, mobile, showing motility; corresponding to the high motility of swimmer cells observed during exponential growth phase).

*Adhaeretor mobilis* exhibits the following properties in addition to those given for the genus: Colonies have a white to cream color and a smooth surface. Growth is preferred in presence of gellan gum compared to agar as solidifying agent. Cells are 1.8 × 1.3 µm in size and react positively for catalase and cytochrome oxidase activity. Growth of the type strain is observed over a temperature range from 12–28 °C with an optimum at 22 °C. The optimal pH for growth is 8.5 while growth was also obtained from pH 5.5 to 10.0. Utilizes a variety of sugar substrates and carboxylic acids, including d-cellobiose, d-galactose, d-glucuronic acid, d-mannitol, d-mannose, d-melibiose, d-psicose, d-trehalose, d,l-lactic acid, gentiobiose, inosine, L-fucose, lactulose, maltose, *N*-acetyl-d-galactosamine, *N*-acetyl-d-glucosamine, sucrose, turanose, α-d-glucose, α-d-lactose, and β-methyl-d-glucoside. The G+C content of the DNA is 55.1%. The type strain is HG15A2^T^ (= VKM B-3444^T^) and was isolated from the epiphytic biofilm community of brown algae of the genus *Laminaria*. 

### 4.3. Updated Description of Roseimaritima Bondoso et al. 2016

Properties of the genus are as given previously [29], with the following amendment: Cells of species belonging to the genus are spherical, ovoid, or have a tear- or drop-like cell shape.

### 4.4. Description of Roseimaritima multifibrata sp. nov.

*Roseimaritima multifibrata* (mul.ti.fi.bra’ta. L. masc. adj. *multus* much, many, numerous; L. fem. adj. *fibrata* fibrous; N.L. fem. adj. *multifibrata* corresponding to the numerous fibers of the cells).

Cells have an average size of 2.0 × 1.0 µm and an elongated tear shape. Cells form multicellular rosettes and aggregates. Motility and spore formation were not observed. Cells divide by polar budding. Extracellular matrix and biofilms are produced during cultivation in liquid culture. Cells form a holdfast structure and crateriform structures were observed at the cell poles. Fimbriae originate from one of the poles. Colonies are pink. Growth is aerobic, mesophilic, and neutrophilic. Growth of the type strain was observed over a temperature range of 12–30 °C with optimal growth at 26 °C. Optimum pH for growth is 7.5 while a pH in the range of 5.5 to 10.0 still allowed for growth. Cells have catalase and cytochrome oxidase activity. The major cellular fatty acid of the type species is C_18:1_ω9c. A variety of sugar substrates and carboxylic acids are degraded: acetic acid, d-fructose, d-galactonic acid lactone, d-galactose, d-galacturonic acid, d-glucuronic acid, d-mannitol, d-melibiose, d-raffinose, d-trehalose, d,l-lactic acid, dextrin, gentiobiose, glucuronamide, L-arabinose, L-fucose, L-rhamnose, lactulose, maltose, methyl pyruvate, *N*-acetyl-d-galactosamine, *N*-acetyl-d-glucosamine, sucrose, turanose, α-d-glucose, α-d-lactose, and β-methyl-d-glucoside. The G+C content of the DNA is 54.5%. The type strain is FF011L^T^ (=DSM 29513^T^ = LMG 29016^T^) and was isolated from the epiphytic biofilm community of brown algae of the genus *Laminaria*.

### 4.5. Description of Rosistilla ulvae sp. nov.

*Rosistilla ulvae* (ul’vae. N.L. gen. n. *ulvae* of *Ulva*, the genus name of the sea lettuce host; corresponding to the origin of the strain).

The cell shape resembles elongated drops or tears. Cells have an average size of 2.4 × 1.2 µm, which form multicellular rosettes and aggregates. Cells are motile and do not form spores. Division takes place by polar budding, with the daughter cell emerging from the bigger pole. Extracellular matrix and biofilm formation in liquid culture are observed during cultivation. Colonies are red and have a smooth surface. Cells show catalase and cytochrome oxidase activity. The major cellular fatty acid is C_16:0_. The type strain grows aerobically over a temperature range of 10–33 °C with optimal growth at 30 °C. Optimal growth was observed at pH 8.0, while growth was also observed over a pH range from 5.5 to 9.5. *Rosistilla ulvae* utilizes a variety of sugar substrates and carboxylic acids, including d-cellobiose, d-fructose, d-galactose, d-glucuronic acid, d-mannitol, d-mannose, d-melibiose, d-psicose, d-raffinose, d-trehalose, d,l-lactic acid, gentiobiose, glucuronamide, glycerol, lactulose, maltose, methyl pyruvate, mono-methyl-succinate, *N*-acetyl-d-galactosamine, *N*-acetyl-d-glucosamine, sucrose, turanose, α-d-glucose, α-d-lactose, and β-methyl-d-glucoside. The G+C content of the DNA is 57.9%. The type strain is EC9^T^ (=DSM 29815^T^ = LMG 29013^T^) and was isolated from the epiphytic biofilm community of green algae of the genus *Ulva*.

### 4.6. Description of Rubripirellula lacrimiformis sp. nov.

*Rubripirellula lacrimiformis* (la.cri.mi.for’mis. L. fem. n. *lacrima* a tear; L. suff. adj. *formis* a form, a figure; N.L. fem. adj. *lacrimiformis* shaped like a tear; corresponding to the tear-shaped morphology of individual cells).

Cells have a tear-shaped morphology and a typical size of 2.5 × 1.4 µm. Cells form multicellular rosettes and aggregates. Motility and spore formation were not observed. Cells divide by polar budding and are pear-shaped. Extracellular matrix and biofilms are produced during cultivation in liquid culture. Cells lack a stalk or holdfast structure and crateriform structures were not observed. Colonies are pink and have a smooth surface. Growth is aerobic, mesophilic and neutrophilic. Growth of the type strain was observed over a temperature range of 10–37 °C with optimal growth at 26 °C. Optimum pH for growth is 8.5 while a pH in the range of 5.5–10.0 still enabled growth. Cells have catalase and cytochrome oxidase activity. The major cellular fatty acid of the type species is C_18:1_ω9c. A variety of sugar substrates and carboxylic acids are degraded: acetic acid, bromo succinic acid, d-galacturonic acid, d-psicose, d-raffinose, glucose-6-phosphate, l-arabinose, methyl pyruvate, *N*-acetyl-d-galactosamine, *N*-acetyl-d-glucosamine, succinic acid, and β-methyl-d-glucoside. The G+C content of the DNA is 57.3%. The type strain is K22.7^T^ (=DSM 29813^T^ = LMG 29017^T^) and was isolated from the epiphytic biofilm community of brown algae of the genus *Fucus*.

## Figures and Tables

**Table 1:** Morphological, physiological and chemotaxonomic characteristics of the novel strains.

Feature	K22.7^T^	FF011L^T^	EC9^T^	HG15A2^T^
Arrangement of cells	Rosettes and aggregates	Rosettes and aggregates	Rosettes and aggregates	Rosettes and aggregates
Cell size (µm)	2.5 ± 0.4 × 1.4 ± 0.3	2.0 ± 0.3 × 1.0 ± 0.2	2.4 ± 0.3 × 1.2 ± 0.2	1.8 ± 0.2 × 1.3 ± 0.2
Cell shape	pear	pear	elongated pear	egg
Isolation source	*Fucus* sp. biofilm	*Laminaria* sp. biofilm	*Ulva* sp. biofilm	*Laminaria* sp. biofilm
Isolation method	Biofilm plating	Floating filter assay	Enrichment cultivation	Biofilm plating
Colony color	pink	pink to red	red	cream
Respiration	aerobic	aerobic	aerobic	aerobic
Oxidase activity	+	+	+	+
Catalase activity	+	+	+	+
Temperature range (°C)	10–37	12–30	10–33	12–28
T_opt_ (°C)	26	26	30	22
pH range	5.5–10.0	5.5–10.0	5.5–9.5	5.5–10.0
pH_opt_	8.5	7.5	8.0	8.5
Growth rate at T_opt_	0.053	0.049	0.062	0.018
Generation time (h) at T_opt_	13.0	14.1	11.1	38.7
Major fatty acid component (%)	C_18:1_ ω9c (42.5)	C_18:1_ ω9c (55.1)	C_16:0_ (37.3)	C_18:1_ ω9c (34.8)

## Supplementary Materials

The following are available online at https://www.mdpi.com/article/10.3390/microorganisms9071494/s1, Figure S1: Phylogeny of the order *Pirellulales* based on whole genome-based multilocus sequence analysis (MLSA), Figure S2: Different parameters used to assign strain K22.7^T^ to the genus *Rubripirellula*, Figure S3: Different parameters to attribute strain FF011L^T^ to the genus *Roseimaritima*. Figure S4. Different parameters to attribute strain EC9^T^ to the genus *Rosistilla*. Figure S5: Different parameters to attribute strain HG15A2^T^ to the family *Lacipirellulaceae*. Table S1: Cultivation media used in this study, Table S2: Oligonucleotides for amplicon generation used in this study, Table S3: Input and output data of the 16S rRNA gene amplicon analysis. Table S4: Cellular fatty acid contents (%) of the novel strains and corresponding closely related type strains, Table S5: Genome characteristics of strain K22.7^T^ and other members of the genus *Rubripirellula*, Table S6: Genome characteristics of strain FF011L^T^ and other members of the genus *Roseimaritima*, Table S7: Genome characteristics of strain EC9^T^ and other members of the genus *Rosistilla*, Table S8: Genome characteristics of strain HG15A2^T^ and other members of the family *Lacipirellulaceae*, Table S9: Secondary metabolite biosynthetic gene clusters determined by AntiSMASH.
