# Supplementary material for: Analysis of Bacterial Communities on North Sea Macroalgae and Characterization of the Isolated Planctomycetes Adhaeretor mobilis gen. nov., sp. nov., Roseimaritima multifibrata sp. nov., Rosistilla ulvae sp. nov. and Rubripirellula lacrimiformis sp. nov
Source: Microorganisms. 2021 Jul 13;9(7):1494. doi: 10.3390/microorganisms9071494 (PMC8303584; doi:10.3390/microorganisms9071494)
Supplement: Supplementary file 1 [file microorganisms-09-01494-s001.zip › microorganisms-1265042-supplementary.pdf]

Supplementary Material

Analysis of bacterial communities on North Sea macroalgae and characterization of the isolated planctomycetes *Adhaeretor mobilis* gen. nov., sp. nov., *Roseimaritima multifibrata* sp. nov., *Rosistilla ulvae* sp. nov. and *Rubripirellula lacrimiformis* sp. nov.

Sandra Wiegand[1,2,#] and Patrick Rast[3,#], Nicolai Kallscheuer[1,4], Mareike Jogler[5], Anja Heuer[3], Christian Boedeker[3], Olga Jeske[3], Timo Kohn[1], John Vollmers[2], Anne-Kristin Kaster[2], Christian Quast[6], Frank Oliver Glöckner[7], Manfred Rohde[8] and Christian Jogler[1,5,*]

[1] Department of Microbiology Radboud University Nijmegen, The Netherlands
[2] Institute for Biological Interfaces 5 (IBG5) Karlsruhe Institute of Technology Karlsruhe, Germany
[3] Leibniz Institute DSMZ Braunschweig, Germany
[4] Institute of Bio- and Geosciences, IBG-1: Biotechnology, Forschungszentrum Jülich GmbH, Jülich, Germany
[5] Department of Microbial Interactions Insitute of Microbiology Friedrich-Schiller University Jena, Germany
[6] Max Planck Institute for Marine Microbiology Bremen, Germany
[7] Alfred Wegener Institute Bremerhaven MARUM University of Bremen, Germany
[8] Central Facility for Microscopy Helmholtz Centre for Infection Research Braunschweig, Germany

* correspondence: email: christian.jogler@uni-jena.de, Tel: +493641949301, # authors contributed equally

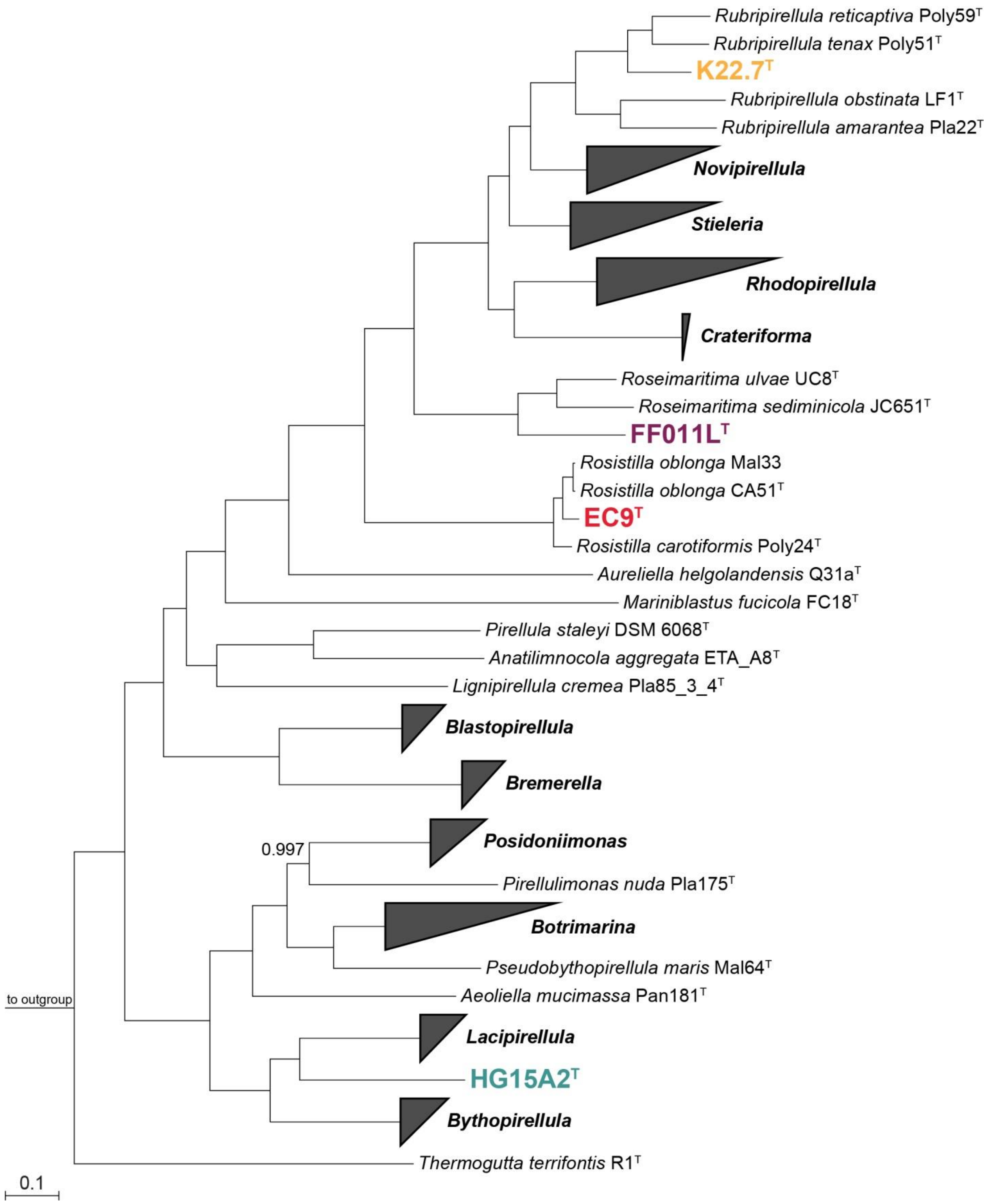

Figure S1. Phylogeny of the order *Pirellulales* based on whole genome-based multilocus sequence analysis (MLSA). Reliability estimators based on Shimodaira-Hasegawa testing were determined. They are only shown in the tree when <1. The outgroup contains three genomes from the family *Planctomycetaceae*.

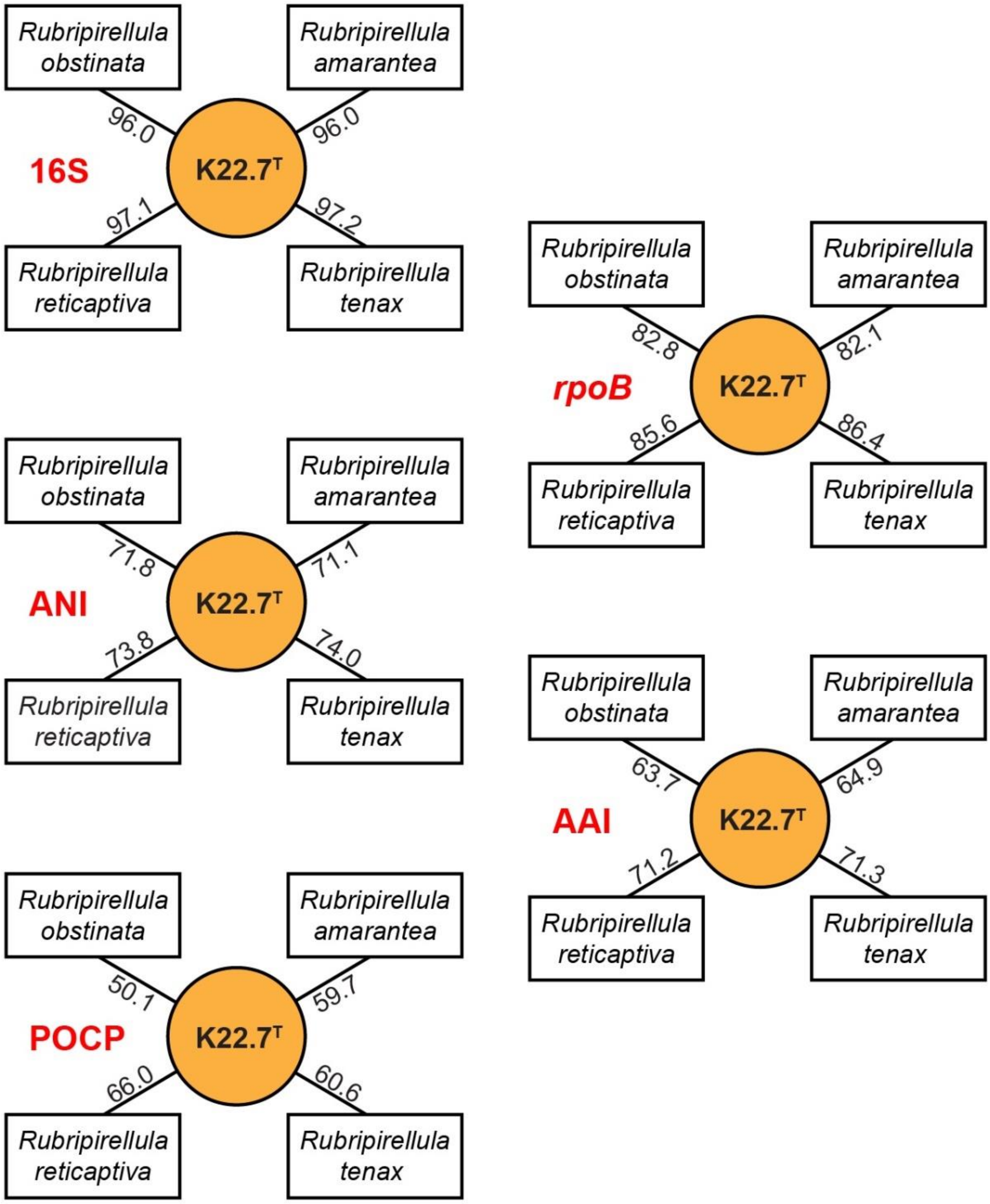

Figure S2. Different parameters used to assign strain K22.7^T to the genus *Rubripirellula*. Identity values were determined for 16S rRNA sequence identity, *rpoB* gene identity, average nucleotide identity (ANI) as well as amino acid identity (AAI) and percentage of conserved proteins (POCP). All values are given in percent.

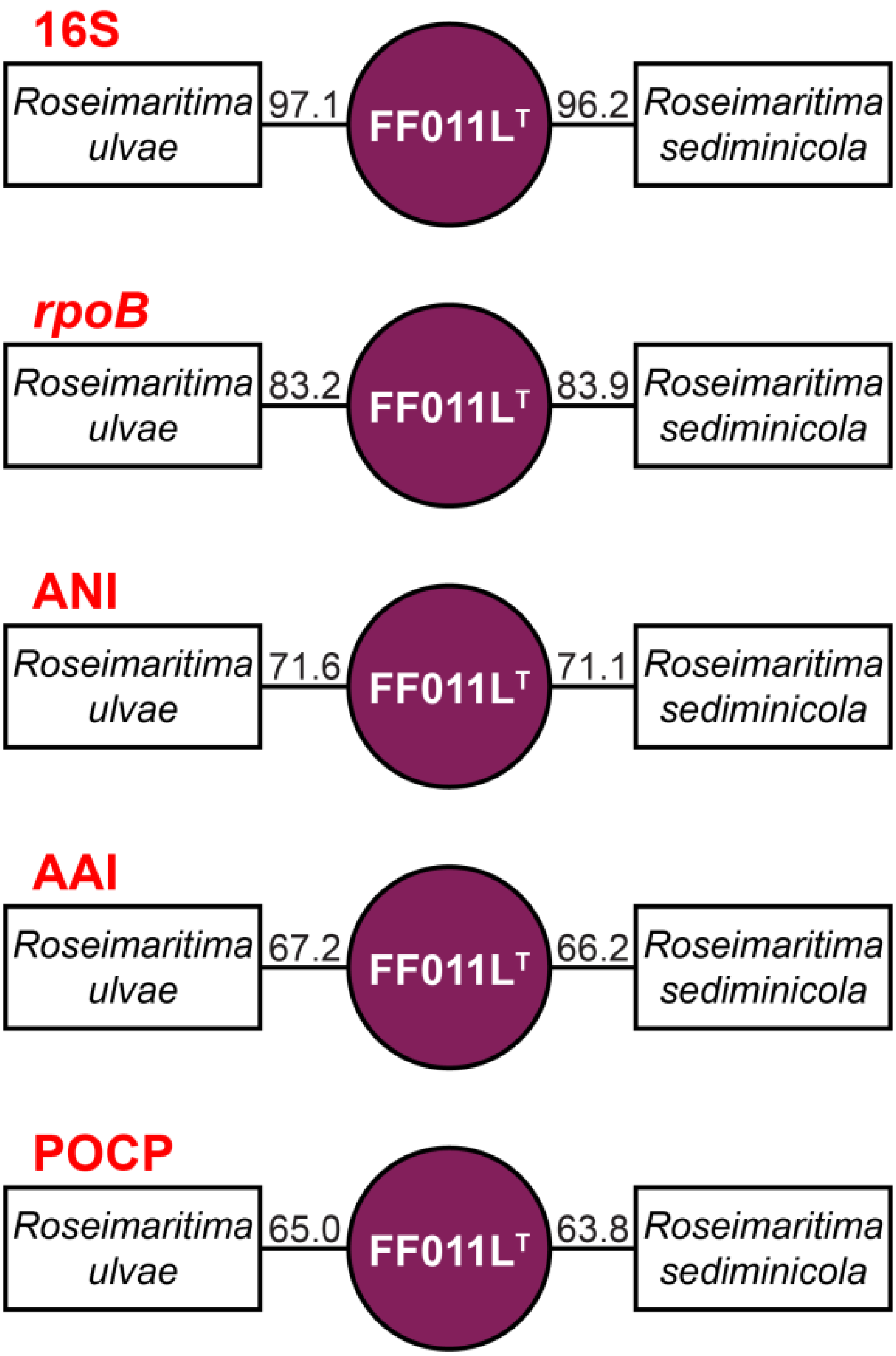

Figure S3. Different parameters to attribute strain FF011L^T to the genus *Roseimaritima*. Identity values were determined for 16S rRNA sequence identity, *rpoB* gene identity, average nucleotide identity (ANI) as well as amino acid identity (AAI) and percentage of conserved proteins (POCP). All values are given in percent.

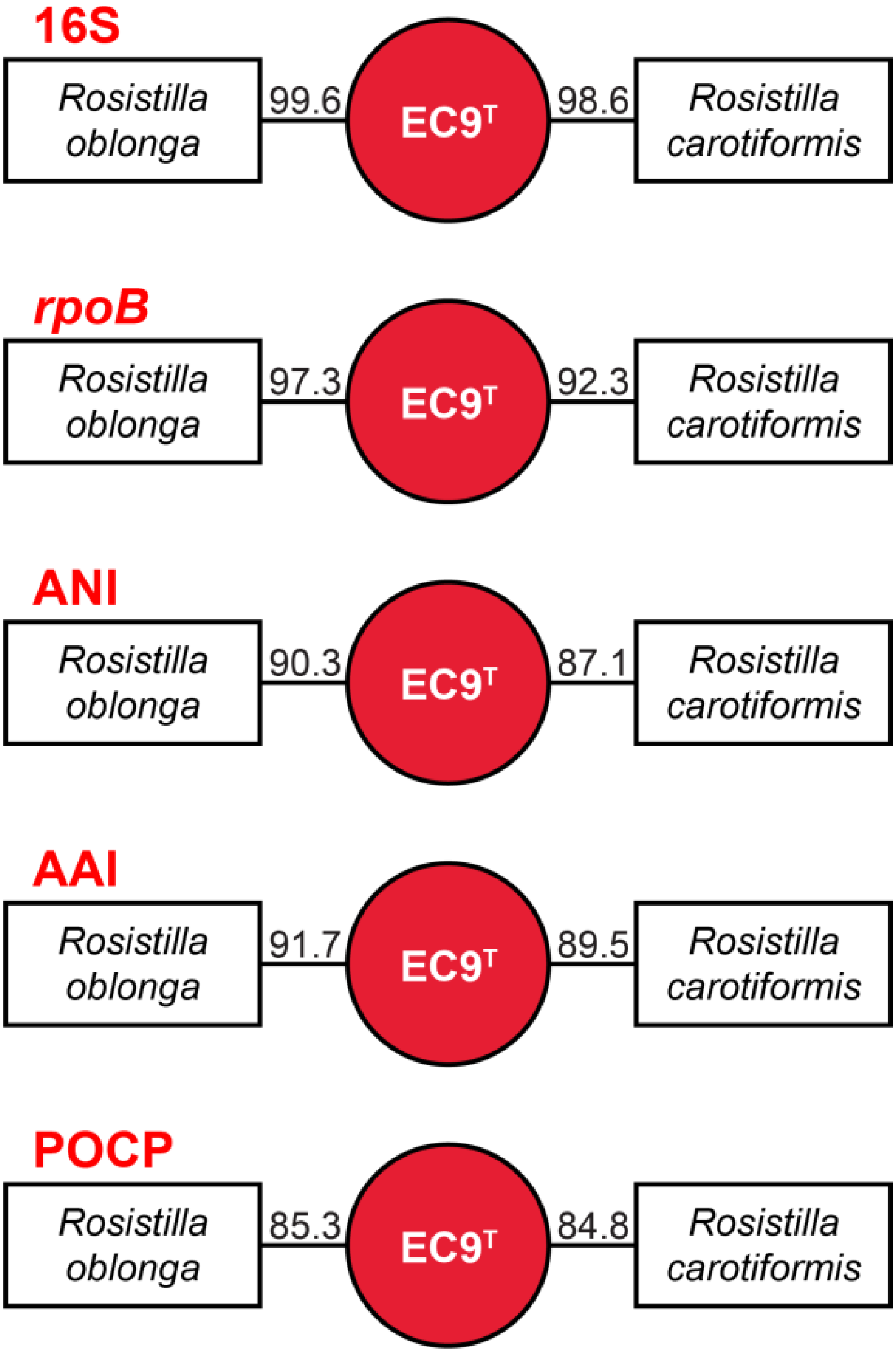

Figure S4. Different parameters to attribute strain EC9^T to the genus *Rosistilla*. Identity values were determined for 16S rRNA sequence identity, *rpoB* gene identity, average nucleotide identity (ANI) as well as amino acid identity (AAI) and percentage of conserved proteins (POCP). All values are given in percent.

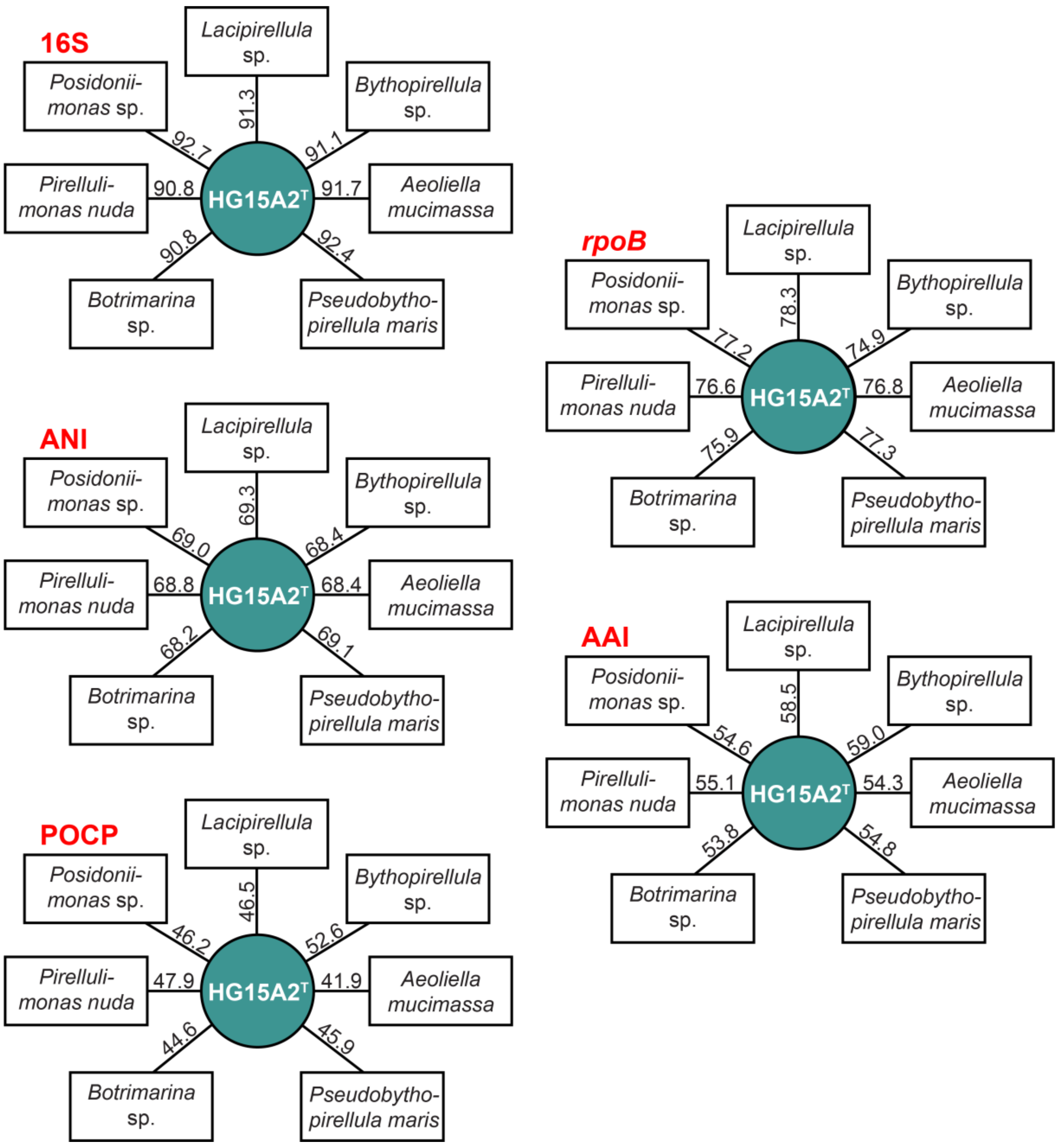

Figure S5. Different parameters to attribute strain HG15A2^T to the family *Lacipirellulaceae*. Identity values were determined for 16S rRNA sequence identity, *rpoB* gene identity, average nucleotide identity (ANI) as well as amino acid identity (AAI) and percentage of conserved proteins (POCP). All values are given in percent.

Table S1. Cultivation media used in this study. (P) peptone, (YE) yeast extract, (NAG) *N*-acetyl-D-glucosamine, (C) carbenicillin, (A/S) ampicillin/streptomycin, (*F. s.*) *Fucus serratus* powder, (G) glucose solution (10 ml/L).

Medium	Purpose	Carbon/nitrogen sources	Fungicide concentration [mg/l]	Antibiotic agent
M1H ASW	Isolation	P, YE, G(10)	0, 20, 100	C or A/S
NAGH ASW	Isolation	NAG	0, 20, 100	C or A/S
M1H NAG ASW	Growth	P, YE, NAG, G(10)	-	-
AMS	Algal attachment	*F. s.*	-	-

Table S2. Oligonucleotides for amplicon generation used in this study. All primers were developed based on a previous study [1]. Each sample was prepared with a unique reverse primer.

Primer	Amplicon	Index sequence [5'→3']
V3R_81	*Laminaria* biofilm	TCT CGG
V3R_92	*Laminaria* glass fibre filter	CGG TGT
V3R_82	*Laminaria* polycarbonate filter	TCA GCG
V3R_32	*Fucus* biofilm	TTG TGA
V3R_36	*Fucus* glass fibre filter	TAA CTC
V3R_33	*Fucus* polycarbonate filter	TTG ACT
V3R_37	*Ulva* biofilm	TAC CAA
V3R_61	*Ulva* glass fibre filter	CAT CTT
V3R_60	*Ulva* polycarbonate filter	CTA GAA

Table S3. Input and output data of the 16S rRNA gene amplicon analysis. OTU: operational taxonomic unit.

	**Number of Sequences**	**Average Length (bp)**	**Total number of OTUs**	**OTUs (phylum *Planctomycetes*)**
Biofilm				
Fucus sp.	32,381	146	546	72
Laminaria sp.	129,618	149	733	4
Ulva sp.	83,580	150	669	22
Seawater particles				
Fucus sp.	40,405	145	866	32
Laminaria sp.	47,671	149	1103	40
Ulva sp.	60,244	151	981	13
Seawater				
Fucus sp.	48,097	149	1252	44
Laminaria sp.	133,343	151	1020	13
Ulva sp.	*57,263*	*150*	*787*	*7*

Table S4. Cellular fatty acid contents (%) of the novel strains and corresponding closely related type strains.

	**K22.7^T**	***Rubripirellula obstinata***	**FF011L^T**	***Roseimaritima ulvae***	**EC9^T**	***Rhodopirellula baltica***	**HG15A2^T**	***'Bythopirellula goksoyri'***
$C_{10:0}$	-	-	-	-	-	-	-	-
$C_{16:0}$	**27.68**	**17.3**	**22.77**	**35.1**	**37.25**	**39.2**	**23.2**	**22.55**
$C_{17:0}$	-	4.5	-	2.1	1.39	1.2	-	1.47
$C_{18:0}$	2.06	5.6	2.52	3.1	7.05	4.3	8.87	**15.32**
$C_{16:1}$ ω9c	-	-	-	-	-	8	-	**26.14**
$C_{16:1}$ ω11c	-	-	-	-	3.28	-	1.34	-
$C_{17:1}$ ω8c	1.89	**12**	4.03	2	-	-	-	-
$C_{17:1}$ ω9c	-	-	-	-		4	-	2.74
$C_{18:1}$ ω7c	1.43	-	4.88	-	-	-	5.84	-
$C_{18:1}$ ω9c	**42.5**	**41.5**	**55.07**	**47**	8.91	**40.8**	**34.77**	**20.73**
$C_{18:1}$ ω11c	-	-	-	-	-	1.6	-	6.23
$C_{20:0}$ ω9c	-	-	1.13	-	-	-	-	-
Iso-$C_{14:0}$	-	-	-	-	-	-	1.82	-
Iso-$C_{16:0}$	-	-	-	-	-	-	2.97	2.65
$C_{12:0}$ 3-OH	-	-	-	-	-	-	1.01	-
$C_{14:0}$ 2-OH	1.39	-	-	-	-	-	-	-
$C_{16:0}$ 2-OH	2.72	1.1	-	-	-	-	-	-
$C_{16:0}$ 3-OH	-	-	-	-	-	-	-	-
$C_{18:0}$ 2-OH	-	-	-	-	-	-	3.5	-
$C_{18:0}$ 3-OH	-	1.3	2.84	-	-	-	-	-
$C_{16:1}$ ω7c/Iso-$C_{15:0}$ 2-OH	**19.26**	-	3.45	-	-	-	**14.69**	-
$C_{18:2}$ ω6,9c/Anteiso-$C_{18:0}$	-	-	-	-	**35.06**	-	-	-

Table S5. Genome characteristics of strain K22.7^T and other members of the genus *Rubripirellula*. The novel strain K22.7^T was compared to strains published before [2, 3]. The genomes were published by [4]. All values were determined based on calculations from CheckM v1.1.2 [5] and Prokka v1.14 [6].

	**K22.7^T**	***Rubripirellula reticaptiva* Poly59^T**	***Rubripirellula tenax* Poly51^T**	***Rubripirellula amarantea* Pla22^T**	***Rubripirellula obstinata* LF1^T**
**Genome size (Mb)**	8.54	7.85	7.99	6.95	7.09
**Scaffolds**	1	13	42	9	27
**G+C content (%)**	57.3	54.8	56.2	53.7	54.3
**Genes (per Mb)**	6512 (762)	6165 (785)	6399 (800)	5260 (756)	5942 (838)
**Protein-coding genes (per Mb)**	6461 (756)	6088 (775)	6274 (785)	5182 (745)	5871 (828)
**Hypothetical proteins**	4320	3996	4237	3315	4046
**rRNAs (16S-23S-5S)**	2-2-2	1-1-3	1-1-2	1-1-1	2-1-1
**tRNAs**	44	71	120	74	66
**Giant genes**	12	9	8	9	9
**Transposable elements**	66	13	19	2	59

Table S6. Genome characteristics of strain FF011L^T and other members of the genus *Roseimaritima*. The novel strain FF011L^T was compared to strains published before [2, 7]. The compared genomes were published previously [4, 7]. All values were determined based on calculations from CheckM v1.1.2 [5] and Prokka v1.14 [6].

	**FF011L^T**	***Roseimaritima ulvae* UC8^T**	***Roseimaritima sediminicola* JC651^T**
**Genome size (Mb)**	7.22	8.21	6.25
**Scaffolds**	1	1	185
**G+C content (%)**	54.5	59.1	62.4
**Genes (per Mb)**	5533 (766)	5903 (719)	4564 (730)
**Protein-coding genes (per Mb)**	5482 (759)	5828 (709)	4510 (721)
**Hypothetical proteins**	3547	3735	2773
**rRNAs (16S-23S-5S)**	2-2-2	1-1-1	1-1-1
**tRNAs**	44	71	50
**Giant genes**	4	10	5
**Transposable elements**	41	55	16

Table S7. Genome characteristics of strain EC9^T and other members of the genus *Rosistilla*. The novel strain EC9^T was compared to strains published before [8]. The genomes were published by [4]. All values were determined based on calculations from CheckM v1.1.2 [5] and Prokka v1.14 [6].

	**EC9^T**	***Rosistilla oblonga*** **CA51^T**	***Rosistilla oblonga*** **Mal33**	***Rosistilla carotiformis*** **Poly24^T**
**Genome size (Mb)**	7.48	7.25	7.51	7.44
**Scaffolds**	1	1	1	1
**G+C content (%)**	57.9	58.2	58.1	57.7
**Genes (per Mb)**	5486 (733)	5297 (730)	5538 (737)	5576 (749)
**Protein-coding genes (per Mb)**	5439 (727)	5249 (724)	5490 (731)	5525 (742)
**Hypothetical proteins**	3436	3258	3486	3511
**rRNAs (16S-23S-5S)**	1-1-1	1-1-1	1-1-1	1-1-1
**tRNAs**	43	44	44	47
**Giant genes**	9	6	5	6
**Transposable elements**	30	63	40	18

Table S8. Genome characteristics of strain HG15A2ᵀ and other members of the family *Lacipirellulaceae*. The novel strain HG15A2ᵀ was compared to strains published before [9-12]. The genomes were published in previous studies [4, 10]. All values were determined based on calculations from CheckM v1.1.2 [5] and Prokka v1.14 [6].

	**HG15A2ᵀ**	***Botrimarina pacificensis* Spa11ᵀ**	***Botrimarina colliarenosi* Pla108ᵀ**	***Botrimarina hoheduenensis* Pla111ᵀ**	***Posidoniimonas corsicana* KOR34ᵀ**	***Posidoniimonas polymericola* Pla123aᵀ**	***Aeoliella mucimassa* Pan181ᵀ**	***Lacipirellula parvula* PX69ᵀ**	***Lacipirellula limnantha* I41ᵀ**	***Pirellulimonas nuda* Pla175ᵀ**	***Bythopirellula goksoeyrii* Pr1d**	***Bythopirellula polymerisocia* Pla144ᵀ**	***Pseudobythopirellula maris* Mal64ᵀ**
**Genome size (Mb)**	5.99	5.87	5.27	4.33	6.77	6.26	6.61	6.92	6.78	6.62	6.47	6.14	5.07
**Scaffolds**	1	1	22	18	13	27	1	1	2	1	1	27	6
**G+C content (%)**	55.1	64.1	65.4	63.1	66.7	66.5	58	61.7	62	66.5	52.8	52.9	66.2
**Genes (per Mb)**	4978 (831)	4655 (793)	4225 (801)	3477 (803)	5343 (789)	4929 (787)	5415 (819)	5658 (817)	5626 (829)	5231 (790)	5395 (833)	5123 (834)	3949 (778)
**Protein-coding genes (per Mb)**	4899 (817)	4597 (783)	4168 (790)	3425 (791)	5247 (775)	4869 (777)	5359 (810)	5581 (806)	5538 (816)	5169 (780)	5325 (823)	5041 (821)	3892 (767)
**Hypothetical proteins**	3191	2944	2597	2058	3408	3113	3379	3662	3712	3265	3500	3251	2381
**rRNAs (16S-23S-5S)**	1-1-1	1-1-1	1-1-1	1-1-1	1-1-1	1-1-1	1-1-1	1-1-1	1-1-1	1-1-1	1-1-1	1-1-1	1-1-1
**tRNAs**	75	54	53	48	92	56	52	73	83	58	66	78	53
**Giant genes**	0	1	1	2	2	1	0	1	0	0	0	0	1
**Transposable elements**	60	7	39	21	10	9	181	9	27	100	34	23	6

Table S9. Secondary metabolite biosynthetic gene clusters determined by AntiSMASH. Genome mining by AntiSMASH yielded seven clusters for strain K22.7^T (genome size: 8.54 Mb), seven for strain FF011L^T (genome size: 7.22 Mb), six for strain EC9^T (genome size: 7.48 Mb) and four for strain HG15A2^T (genome size: 5.99 Mb). The number of clusters correlate with the genome size to a certain extent.

	**Predicted compound class**	**Putative annotation of key genes**
**K22.7^T**		
Cluster 1	terpene	phytoene synthase; squalene synthase
Cluster 2	type III polyketide	type III PKS
Cluster 3	terpene	terpene cyclase; polyprenyl synthetase
Cluster 4	non-ribosomal peptide	AMP-dependent synthetase/ligase (NRPS-like)
Cluster 5	acyl amino acid	*N*-acyl amino acid synthase
Cluster 6	non-ribosomal peptide	AMP-dependent synthetase/ligase (NRPS-like)
Cluster 7	mixed polyketide/ non-ribosomal peptide	type I PKS; AMP-dependent synthetase/ligase (NRPS-like)
**FF011L^T**		
Cluster 1	terpene	terpene cyclase; polyprenyl synthetase
Cluster 2	bacteriocin	DUF692 family protein
Cluster 3	non-ribosomal peptide	AMP-dependent synthetase/ligase (NRPS-like); dioxygenase, TauD/TfdA; condensation domain-containing protein
Cluster 4	non-ribosomal peptide	AMP-dependent synthetase/ligase (NRPS-like)
Cluster 5	polyketide	type III PKS
Cluster 6	terpene	phytoene synthase; squalene synthase
Cluster 7	mixed polyketide/ non-ribosomal peptide	type I PKS; AMP-dependent synthetase/ligase (NRPS-like)
**EC9^T**		
Cluster 1	polyketide	type III PKS
Cluster 2	terpene	polyprenyl synthetase; dehydrogenase; phytoene synthase; lycopene cyclase
Cluster 3	non-ribosomal peptide	AMP-dependent synthetase/ligase (NRPS-like)
Cluster 4	non-ribosomal peptide	AMP-dependent synthetase/ligase (NRPS-like) asparagine synthase; alcohol dehydrogenase
Cluster 5	thiopeptide	radical SAM protein; OsmC/YcaO domain-containing protein
Cluster 6	non-ribosomal peptide	2x AMP-dependent synthetase and ligase (NRPS-like); acyl-CoA dehydrogenase; condensation domain-containing protein
**HG15A2^T**		
Cluster 1	other	hypothetical protein - putative LmbU regulator
Cluster 2	polyketide	type I PKS
Cluster 3	non-ribosomal peptide	AMP-dependent synthetase/ligase (NRPS-like)
Cluster 4	terpene	terpene cyclase; polyprenyl synthetase

References

1. Bartram, A. K.; Lynch, M. D.; Stearns, J. C.; Moreno-Hagelsieb, G., and Neufeld, J. D. Generation of multimillion-sequence 16S rRNA gene libraries from complex microbial communities by assembling paired-end illumina reads. *Appl. Environ. Microbiol.* **2011**, 77, 3846-52.

2. Bondoso, J.; Albuquerque, L.; Nobre, M. F.; Lobo-da-Cunha, A.; da Costa, M. S., and Lage, O. M. *Roseimaritima ulvae* gen. nov., sp. nov. and *Rubripirellula obstinata* gen. nov., sp. nov. two novel planctomycetes isolated from the epiphytic community of macroalgae. *Syst. Appl. Microbiol.* **2015**, 38, 8-15.

3. Kallscheuer, N.; Jogler, M.; Wiegand, S.; Peeters, S. H.; Heuer, A.; Boedeker, C.; Jetten, M. S. M.; Rohde, M., and Jogler, C. Three novel *Rubripirellula* species isolated from plastic particles submerged in the Baltic Sea and the estuary of the river Warnow in northern Germany. *Antonie Van Leeuwenhoek* **2020**, 113, 1767-78.

4. Wiegand, S.; Jogler, M.; Boedeker, C.; Pinto, D.; Vollmers, J.; Rivas-Marin, E.; Kohn, T.; Peeters, S. H.; Heuer, A.; Rast, P.; *et al.* Cultivation and functional characterization of 79 planctomycetes uncovers their unique biology. *Nat. Microbiol.* **2020**, 5, 126-40.

5. Parks, D. H.; Imelfort, M.; Skennerton, C. T.; Hugenholtz, P., and Tyson, G. W. CheckM: assessing the quality of microbial genomes recovered from isolates, single cells, and metagenomes. *Genome Res.* **2015**, 25, 1043-55.

6. Seemann, T. Prokka: rapid prokaryotic genome annotation. *Bioinformatics* **2014**, 30, 2068-9.

7. Kumar, D.; Gaurav, K.; U, J.; G, D.; Ch., S., and Ch.V., R. *Roseimaritima sediminicola* sp. nov., a new member of Planctomycetaceae isolated from Chilika lagoon. *Int. J. Syst. Evol. Microbiol.* **2020**, 70, 2616-23.

8. Waqqas, M.; Salbreiter, M.; Kallscheuer, N.; Jogler, M.; Wiegand, S.; Heuer, A.; Rast, P.; Peeters, S. H.; Boedeker, C.; Jetten, M. S. M.; *et al. Rosistilla oblonga* gen. nov., sp. nov. and *Rosistilla carotiformis* sp. nov., isolated from biotic or abiotic surfaces in Northern Germany, Mallorca, Spain and California, USA. *Antonie Van Leeuwenhoek* **2020**, 113, 1939-52.

9. Storesund, J. E., and Ovreas, L. Diversity of Planctomycetes in iron-hydroxide deposits from the Arctic Mid Ocean Ridge (AMOR) and description of *Bythopirellula goksoyri* gen. nov., sp. nov., a novel Planctomycete from deep sea iron-hydroxide deposits. *Antonie Van Leeuwenhoek* **2013**, 104, 569-84.

10. Dedysh, S. N.; Kulichevskaya, I. S.; Beletsky, A. V.; Ivanova, A. A.; Rijpstra, W. I. C.; Damste, J. S. S.; Mardanov, A. V., and Ravin, N. V. *Lacipirellula parvula* gen. nov., sp. nov., representing a lineage of planctomycetes widespread in low-oxygen habitats, description of the family *Lacipirellulaceae* fam. nov. and proposal of the orders *Pirellulales* ord. nov., *Gemmatales* ord. nov. and *Isosphaerales* ord. nov. *Syst. Appl. Microbiol.* **2020**, 43, 126050.

11. Kohn, T.; Rast, P.; Kallscheuer, N.; Wiegand, S.; Boedeker, C.; Jetten, M. S. M.; Jeske, O.; Vollmers, J.; Kaster, A. K.; Rohde, M.; *et al.* The Microbiome of *Posidonia oceanica* Seagrass Leaves Can Be Dominated by Planctomycetes. *Front. Microbiol.* 2020, 11, 1458.

12. Kallscheuer, N.; Rast, P.; Jogler, M.; Wiegand, S.; Kohn, T.; Boedeker, C.; Jeske, O.; Heuer, A.; Quast, C.; Glöckner, F.-O.; *et al.* Analysis of bacterial communities in a municipal duck pond during a phytoplankton bloom and isolation of *Anatilimnocola aggregata* gen. nov., sp. nov., *Lacipirellula limnantheis* sp. nov. and *Urbifossiella limnaea* gen. nov., sp. nov. belonging to the phylum Planctomycetes. *Environ. Microbiol.* **2021**, 23, 1379-96.
